# The effect of spokesperson attribution on public health message sharing during the COVID-19 pandemic

**DOI:** 10.1371/journal.pone.0245100

**Published:** 2021-02-03

**Authors:** Ahmad Abu-Akel, Andreas Spitz, Robert West

**Affiliations:** 1 Institute of Psychology, University of Lausanne, Lausanne, Switzerland; 2 School of Computer and Communication Sciences, Ecole Polytechnique Fédérale de Lausanne, Lausanne, Switzerland; Hong Kong Polytechnic University, HONG KONG

## Abstract

It is urgent to understand how to effectively communicate public health messages during the COVID-19 pandemic. Previous work has focused on how to formulate messages in terms of style and content, rather than on who should send them. In particular, little is known about the impact of spokesperson selection on message propagation during times of crisis. We report on the effectiveness of different public figures at promoting social distancing among 12,194 respondents from six countries that were severely affected by the COVID-19 pandemic at the time of data collection. Across countries and demographic strata, immunology expert Dr. Anthony Fauci achieved the highest level of respondents’ willingness to reshare a call to social distancing, followed by a government spokesperson. Celebrity spokespersons were least effective. The likelihood of message resharing increased with age and when respondents expressed positive sentiments towards the spokesperson. These results contribute to the development of evidence-based knowledge regarding the effectiveness of prominent official and non-official public figures in communicating public health messaging in times of crisis. Our findings serve as a reminder that scientific experts and governments should not underestimate their power to inform and persuade in times of crisis and underscore the crucial importance of selecting the most effective messenger in propagating messages of lifesaving information during a pandemic.

## Introduction

Overcoming public crises may require collective behavior change [1]. Public policy efforts to combat the coronavirus disease 2019 (COVID-19) pandemic focus on social distancing [2], contact tracing [3], and vaccination, all of which can yield the desired results only if they are adopted rapidly by a substantial fraction of the population and sustained for an extended period of time [4]. In order to achieve broad compliance with such measures, communicating with the affected population in a coordinated, effective, and credible way is a key factor [5], and reaching a large audience beyond the initial recipients of a message is paramount. Hence, understanding the factors that result in the most persuasive communication is critical for public-health officials, not just because the world is currently in the grip of one pandemic that is likely to be prolonged [4], but also because it is not too early to start thinking about contingencies for the next pandemic [6].

A large body of work has investigated the question of *how* to frame public messages in order to maximize their persuasiveness [7, 8], and identified as key characteristics evidence-based information [9], message style and content features [10], emphasizing the benefit to the recipient [11], and aligning with the recipient’s moral values [12] and personality [13]. During the 2003 SARS outbreak, best practices and strategies for crisis communication were developed [14], and clarity of speech, openness, and honesty were identified as the most important positive personal characteristics for official spokespersons, while inappropriate demeanor, lack of honesty, poor emotional control, political bias, and bureaucratic style were considered negative characteristics [15].

Beyond the content of a message alone, *who* communicates the message is one of the most important factors in determining its perlocutionary force [16], and the successful serial distribution of warning messages in crisis situations has been linked to a strong first-order exposure [17]. Celebrities in particular have been shown to exert a strong influence on public opinion at large [18, 19], including opinions about health and well-being [20]. Yet, little is known about their effectiveness during times of crisis. Extant research suggests that during *simulated* crises, government officials garner greater support for intervention and interest in the crisis than celebrities [21], and that the public tends to rally around their government during crises [22, 23]. Overall, whereas the problem of choosing the wording of a message (*How* to formulate the message?) has received ample attention, the problem of choosing a spokesperson (*Who* should send the message in order to maximize its effectiveness?) has been largely understudied, especially in times of crisis.

Our work aims to narrow this gap by quantifying the effectiveness of various public figures as advocates for social distancing during the COVID-19 pandemic. In the design of such a study, we necessarily have to consider its placement within the space of *message effectiveness* and *achieved result*, which both are equally important in reaching and convincing a substantial fraction of the population. Possible considerations for message effectiveness are how effective the message is at convincing a recipient to adopt its content (*adoption effectiveness*), as well as how effective it is at reaching a large audience (*redistribution effectiveness*). In our study, we focus on redistribution effectiveness, which we consider a necessary precursor for persuasion on a societal scale, since even the most convincing message has little effect without sufficient distribution. With regard to the achieved result, one may consider the respondents’ stated intent on the one hand (*intent to reshare content*), and subsequently observed action on the other (*content resharing*). In our study, we investigate the effectiveness of spokesperson selection on the stated intent to reshare a received public health message as a proxy for actual resharing, since intent has been linked to tangible behavior in comparable settings [24].

We examined the spokesperson effect on the stated intent to reshare across six countries in which the transmission of COVID-19 was rapidly intensifying at the time of research (Brazil, Italy, South Korea, Spain, Switzerland, United States) and across age groups, given evidence for age-related effects on social distancing compliance and risk perception [1, 25]. In addition to the identity of the spokesperson, we also investigated the effect of their likeability, since prior research has demonstrated that likeability moderates the impact of persuasive messages [26]. There is also evidence showing that an individual may perceive the argument as better or stronger and therefore as more persuasive if they like the source of the message, which may indicate a "likeability" heuristic [27]. We leveraged a survey that, at the surface, aimed to gauge respondents’ perception of the pandemic and their level of compliance with, as well as support for, social distancing measures. The survey was designed as a randomized controlled trial by stating that social distancing had been endorsed, among others, by a certain public figure, who thus served as a spokesperson for social distancing. When a respondent opened the survey, the identity of the spokesperson was drawn randomly from a set of four candidates (see *Materials and methods* for details on the survey and the choice of spokespersons): an immunology expert (Dr. Anthony Fauci), two widely known celebrities (actor Tom Hanks and media personality Kim Kardashian), and an elected government official, who was specific to the respondent’s country (where possible, we used the head of government if they had previously officially endorsed social distancing). Additionally, there was a control condition where social distancing was introduced without mentioning any spokesperson’s endorsement. As the outcome variable, we used the respondent’s stated willingness to share the spokesperson’s endorsement of social distancing on their own social media (henceforth, “message sharing”). The survey was conducted between March 24 and 30, 2020, with participants recruited mainly via social media ads targeting specific demographic groups (*N* = 12,194), as detailed in the following.

## Materials and methods

### Respondents

Data were obtained from 12,575 respondents from six countries in which the transmission of COVID-19 was rapidly intensifying at the time of research: Brazil, Italy, South Korea, Spain, Switzerland, and the United States. We detail selection criteria for respondents under *Data preparation*. The final sample consisted of 12,194 respondents (7,316 females, mean age 37.04 (SD 14.80), range 18–80). S1 Table in S1 File provides characteristics and summary data of the survey overall and by country.

Respondents were mainly recruited through a stratified advertisement campaign on Facebook between March 24 and 30, 2020. The ad consisted of a rendered image of the virus, the sentence “Help us understand how COVID-19 is affecting people’s lives in a 3-minute survey”, and a link that redirected to one of the five spokesperson-specific survey forms (see SD Appendix in S1 File). Participation in the survey was voluntary and not remunerated. The advertisement budget ($3000) was split evenly across the six countries, targeting residents in their native language. Within each targeted country, the campaign was evenly subdivided across eight strata (male/female, as well as the age groups 18–25, 26–40, 41–60, and 61+) and the five spokesperson conditions. Click-through optimization was used as the campaign goal. Parallel to the advertisement campaign, a multilingual website, which redirected visitors to the survey form for a randomly selected spokesperson in a chosen language, was made available and publicized at EPFL and on Twitter.

### Sample representativeness

Since participants were primarily recruited through an advertisement campaign on Facebook, our sample may be subject to sampling bias, as is typically the case for online surveys. To mitigate potential sampling bias effects, we stratified the advertisement campaign by age, gender, and geographic location, and respondents were not remunerated for their participation, as described under *Respondents*. As can be seen from S1 Fig in S1 File, our sample was overrepresented in respondents who are female (60%), young (48.6%), employed/self-employed (59.7%), highly educated (over 14 years of education, 58.5%), and non-urban (living in a village, small town, or town, 61.9%).

### Survey design

The study was designed as a randomized controlled trial in which data were collected through an online survey form. Assignment to trial conditions occurred algorithmically and uniformly at random, and we were blinded to this assignment. For the full content of the English version of the survey, see SA Appendix in S1 File. For each country, the English survey form was translated to the official language(s) by a native speaker. The main outcome measure of the study was the respondents’ intention of sharing a message that recommended the practice of social distancing, which we adapted from the definition by Johns Hopkins Medicine [28]. After being shown this message, respondents were asked how likely they were to share this message on their own social media (Q3). The design of the survey was identical for all respondents, with the exception of (i) mentions of the country and government, which were adapted to the respondent's country of residence, and (ii) the identity of the spokesperson shown to support the social distancing message. Spokesperson support was included immediately after the message about social distancing and consisted of a picture of the spokesperson and a statement reading, “Social distancing has been publicly supported, among others, by *[job description and name of the spokesperson]”*. Respondents were randomly assigned one of four possible spokespersons or a No Speaker condition, in which the statement was not supported by a spokesperson (assignments to one of the five groups were implemented as A/B tests on Facebook Ads, to ensure that participants only ever saw one survey form). Respondents in one of the four groups that included a spokesperson were also asked whether they liked, disliked, were neutral toward, or did not know the spokesperson (Q10).

The four spokespersons were selected to respectively represent (i) a source of official government instructions on social distancing, (ii) a well-known medical expert with a background related to the outbreak, or an unofficial endorsement by an unaffiliated celebrity that had either (iii) contracted COVID-19 or (iv) been personally unaffected. To avoid spreading misinformation at such a crucial time, we ensured that all spokespersons had previously issued public support of social distancing (see SB Appendix in S1 File). As the government spokesperson, we selected the head of state when we could verify, at the time of the survey, their support of social distancing: Donald Trump (United States), Simonetta Sommaruga (Switzerland), Giuseppe Conte (Italy), Pedro Sánchez (Spain), and Moon Jae-in (South Korea). We were unable to find any evidence of support by the President of Brazil, Jair Bolsonaro, and instead used Luiz Henrique Mandetta, the Minister of Health at the time of research, as the spokesperson. As a medical expert spokesperson, we used Dr. Anthony Fauci, due to his expertise in immunology and infectious diseases and his prominent position in the U.S. (and the highest likelihood of being known worldwide). As celebrity speakers, we selected Tom Hanks (who had contracted COVID-19 prior to the survey and attracted media attention for his endorsement of social distancing) and Kim Kardashian (who had been highly outspoken about social distancing). Both are well-known across age groups and to an international audience, which made them likely to be known by respondents in all six countries, for which we found the selection of comparable local celebrities infeasible. In the selection of images for spokespersons, we ensured that images were of the same high quality, showed no other persons, no confusing or overly colorful background, no national symbols or flags in the background, and that the spokespersons were facing the camera with a neutral facial expression.

To establish a baseline of respondents’ views and attitudes, the survey also elicited responses to determine the extent to which respondents were aware of, and showed support for, social distancing (Q4, Q5, and Q6), currently practiced social distancing (Q7), intended to practice social distancing in the future (Q9), and the degree to which they perceived others practicing social distancing (Q8). Furthermore, we elicited participants’ worry about the current situation as a whole (Q1) and for the well-being of their fellow citizens (Q12), as well as the perceived spread of the pandemic in their community (Q11). In addition, the following demographic and attitudinal variables were collected: age (Q18), gender (Q17), employment status (Q20), years of education (Q19), household size (Q23), settlement size (village, small town, town, city, metropolitan area; Q22), general subjective health (Q13), religiosity (Q24), perceived freedom of movement (Q14), satisfaction with their government’s efforts to combat COVID-19 (Q15), and perception of their government’s concern for public health versus the economy (Q16).

### Study in context

**Fig 1:**
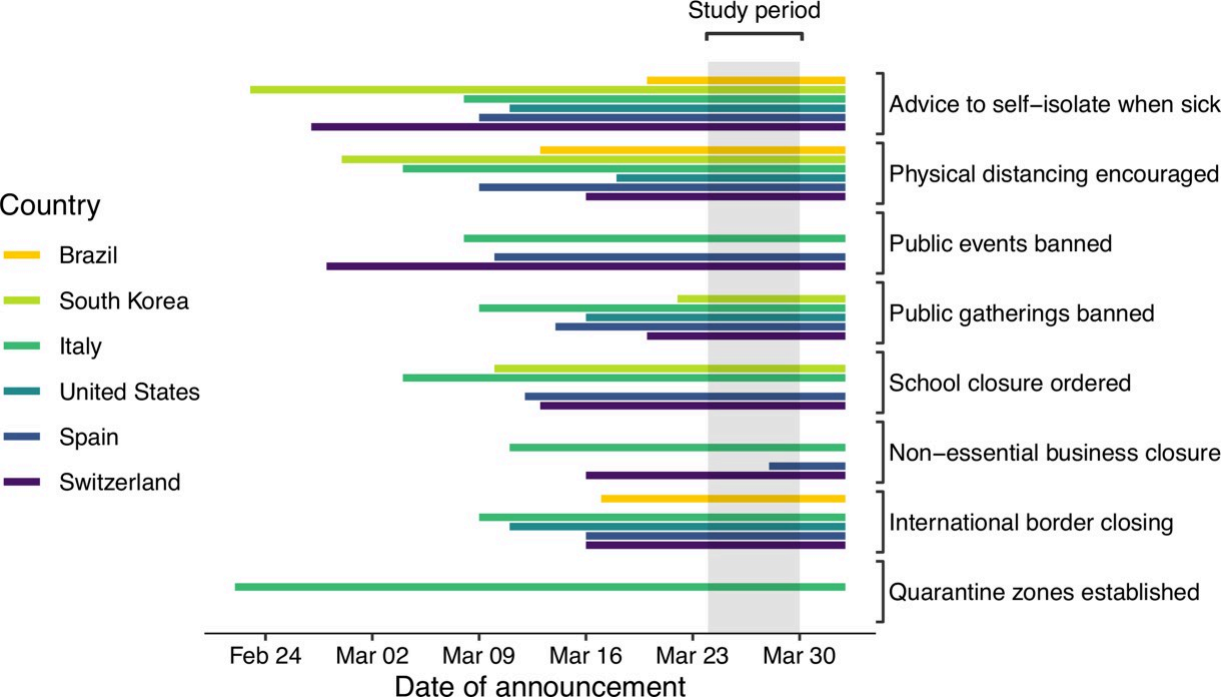
Temporal context of the study. Shown are the dates at which key social distancing measures were announced on a national level by countries in the study. The time frame of data collection (March 24–30, 2020) is highlighted in gray. Empty bars indicate that no action was announced or taken by the national government (for comparability between federal states and unitary states, we only considered announcements by the federal government in federated countries, even though there may have been actions on a local, city, or state level). For a detailed list of these government announcements, see SC Appendix in S1 File.

The survey was administered during the period of March 24–30, 2020, two weeks after COVID-19 had been declared a pandemic by the WHO on March 11 [29]. From February 22, when Italy first established quarantine zones around twelve severely affected regions in Lombardy, a number of social and physical distancing measures were progressively introduced by the countries in our study (see Fig 1; SC Appendix in S1 File). Advice to keep physical distance at all times and to self-isolate at home when suffering from respiratory problems that could be linked to the virus had been formally issued by the national governments of all six countries at least a few days prior to the start of the survey. With the exception of Brazil, public gatherings had been banned or discouraged in all countries by issuing stay-at-home orders or lockdowns. More drastic measures, including the mandated cancelation of public events and the closure of non-essential businesses (in Italy, Spain, and Switzerland), as well as the closure of schools and universities (also implemented in South Korea in addition to the former three), were enforced only by some of the countries on a national level. In the remaining countries, these measures had also been used by the start of our survey period, but only on a local or state level and without support of the national or federal government. The government-mandated shutdown of non-essential businesses in Spain is the only measure for which the announcement coincided with our survey period. The closure of some or all international borders to non-residents was implemented by all countries with the exception of South Korea, which instead enforced strict quarantine and testing protocols upon arrival. Quarantine zones were only implemented in Italy during the early phase of the outbreak and effectively overridden by the country-wide lockdown on March 9.

### Ethical compliance

This research is part of a larger project for which ethical approval has been obtained from the EPFL Human Research Ethics Committee. All survey participants were informed that their responses would be used as part of a research project prior to the submission of the survey, and the submission was regarded as consent.

### Data and materials availability

The full data that were generated and analyzed during the study, as well as the computer code that was generated for the study, are available on Github at https://github.com/epfl-dlab/SpokespersonAttributionCOVID.

### Data preparation

We intended to collect data from adults between age 18 and 80 to maximize data reliability, which is recommended for online surveys [30]. We therefore excluded 326 respondents whose reported age fell outside this range (Age < 18, *N* = 296; Age > 80, *N* = 30). We also excluded 55 respondents who specified “Other” as their gender. In addition, 118 “household size” entries that equaled zero or had a value greater than 15 were considered invalid, but not removed. The values were imputed with the mean of the valid data entries. In total, we excluded 381 outliers out of 12,575 data points. No participants dropped out of the study, and the analyses were based on the remaining 12,194 respondents.

### Age distribution and clustering

Due to the non-unimodal structure of the age distribution of our sample (Hartigans’ dip test (D_5000_) = 0.026, *p* < 2.2x10^-16^), we performed a 2-step cluster analysis, using Schwarz’ Bayesian criterion, to identify potential subgroups. A 3-cluster solution (see S2 Fig in S1 File) was deemed optimal with a silhouette score of 0.7 (a measure of “cohesion and separation” of clusters), suggesting a good cluster structure [31]. The three age groups were characterized as young (*N* = 5931, range 18–32 years), mid-age (*N* = 3618, range 33–50 years) and old (*N* = 2645, range = 51–80 years). S2 Table in S1 File provides age and gender distributions of the three age groups by country and in the overall sample.

### Statistical analyses

First, we measured Spearman’s correlation between the study’s variables, with Bonferroni correction. The main analyses were performed using *Generalized linear mixed models with robust estimations in SPSS 25*. In a linear regression, we examined the main effect of spokesperson, age group, and country, the 2-way interactions of spokesperson × country and spokesperson × age group, and the 3-way interaction of spokesperson × country × age group on message sharing. The model was fitted while controlling for the following demographic and attitudinal measures by adding them as regression terms: age, gender, employment status, years of education, household size, settlement size, subjective health, perceived fraction of population infected by coronavirus, level of concern about COVID-19, concern for the well-being of others, perception of others’ practice of social distancing, religiosity, liberty of movement, satisfaction with government efforts to combat COVID-19, and perception of the government’s concern for public health versus the economy. In addition, we controlled for the number of social distancing measures they endorsed (from a list of nine measures, see Q6 in SA Appendix in S1 File), the extent to which respondents supported social distancing, currently practiced social distancing, and intended to practice social distancing in the future. We control for these various attitudinal and demographic variables because studies that investigated responses during the early stages of this pandemic as well as prior pandemics have shown that compliance can be affected by a number of important demographic (e.g., age, gender), attitudinal (e.g., perceived health status, attitudes towards public health and government officials) [5, 32], and psychological factors such as risk perception and concern for others [32–34]. While randomization is likely to reduce the impact of controlling for these variables, any randomized control trial with finite size will suffer from some degree of imbalance in residual covariance, and so we follow standard practice and account for this fact by controlling for these attitudinal and demographic variables. We cannot rule out self-selection as a result of the treatment step (participants may be more likely to submit the surveys for some spokespersons than for others).

In addition, to examine if the above effects on message sharing varied by the respondents’ sentiments towards the spokespersons (namely, towards Fauci, Government, Hanks and Kardashian), we repeated the same analysis by adding the likeability factor and examined the main effect of spokesperson, country, and likeability, the 2-way interactions of spokesperson × country, country × likeability, spokesperson × likeability, and the 3-way interaction of spokesperson × country × likeability. For this analysis, the outcome measure was the standardized residual of the message sharing scores, adjusted for all demographic and attitudinal measures mentioned above. Moreover, in a separate linear regression, we also computed the standardized residual of the message sharing scores under the no-spokesperson condition, by partialing out all demographic and attitudinal measures mentioned above. This was performed in order to be able to compare the relative effect of the four spokespersons, under the different likeability categories, to the no-spokesperson condition, for which a likeability could not be elicited (see Fig 6A).

**Fig 2:**
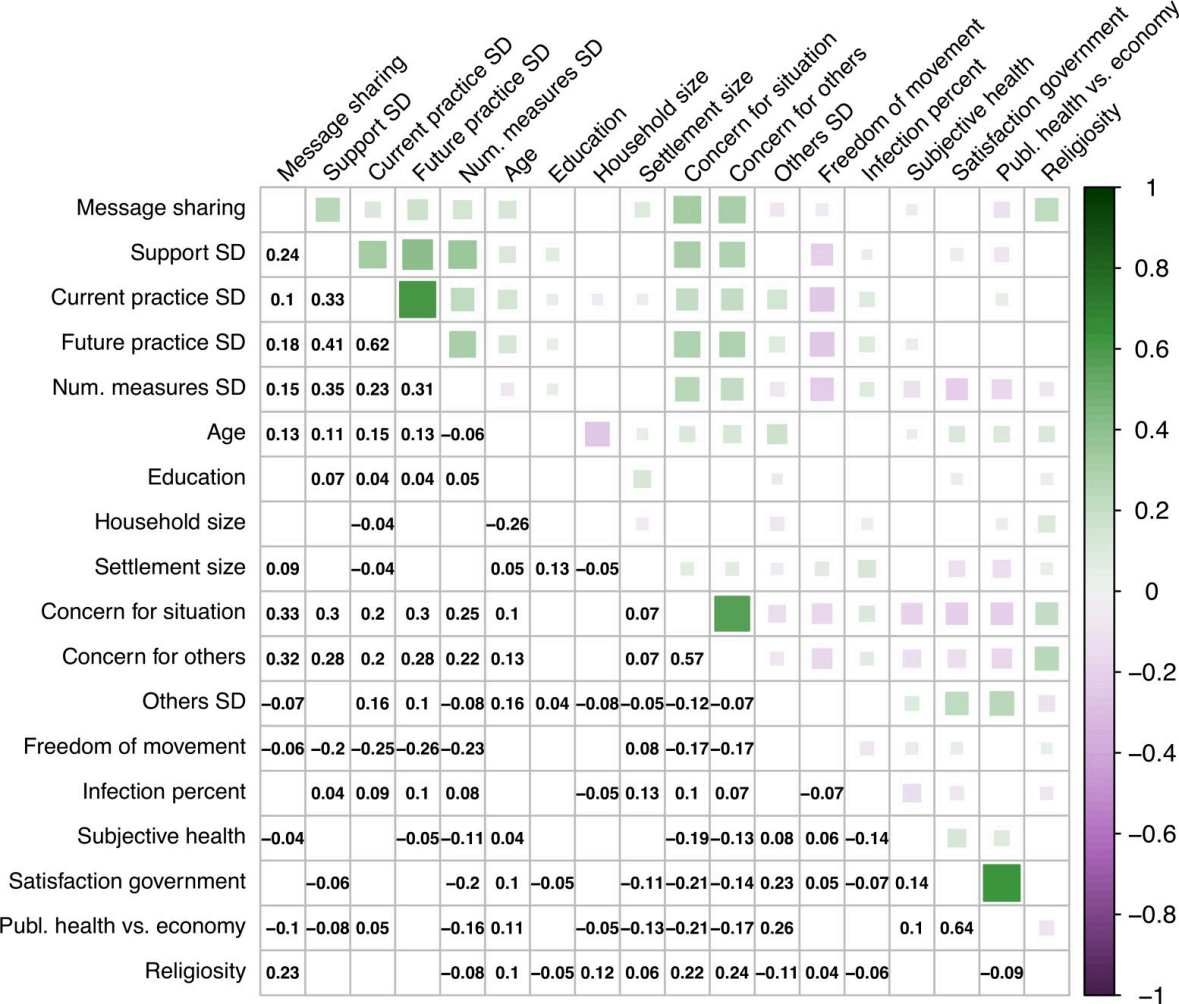
Correlation matrix of all study variables. Significance threshold is Bonferroni-corrected, *p* < 3.3x10^-4^. Empty cell = non-significant correlation; SD = Social distancing; Num. = Number.

All pairwise comparisons were subjected to sequential Bonferroni correction. For the correlation matrix (Fig 2), we applied the more conservative Bonferroni correction. Effect sizes are reported in terms of Cohen’s *d* (in absolute values) and partial eta squared (η_p_^2^).

## Results

First, to estimate if the self-reported declaration of willingness to share the message (henceforth referred to as message sharing) was associated with demographic and attitudinal measures, we calculated bivariate correlations. Spearman’s correlations revealed that message sharing was significantly associated with a number of demographic and attitudinal measures (Fig 2, all *p* < 3.3x10^-4^, Bonferroni-corrected for multiple testing). Specifically, message sharing was positively associated with support for social distancing (*r* = 0.24), current practice of social distancing (*r* = 0.10), the intention to practice social distancing in the future (*r* = 0.18), and the total number of endorsed social distancing measures (*r* = 0.15) (see also S3 Fig in S1 File, which examines in more detail the pattern of endorsement of nine social distancing measures [see Q6 in SA Appendix in S1 File] by spokesperson and country). It was also positively associated with age (*r* = 0.13), concern for the situation (*r* = 0.33), concern for others (*r* = 0.32), settlement size (*r* = 0.09), and religiosity (*r* = 0.23). It was negatively correlated with the perception that others are practicing social distancing (*r* = -0.07), greater freedom of movement (*r* = -0.06), better subjective health (*r* = -0.04), and the perception that the government prioritizes public health over the economy (*r* = -0.10).

**Fig 3:**
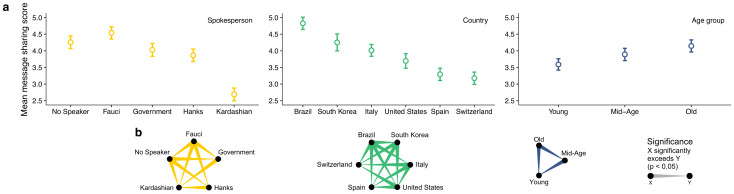
Main effects of spokesperson, country and age group on message sharing. **(a)** Message sharing score on a 1–7 Likert scale. Error bars represent 95% CIs. **(b)** Corresponding, color-coded significant pairwise comparisons, accounting for multiple comparisons via sequential Bonferroni correction. Only significant comparisons are shown. The results show that the medical spokesperson Dr. Fauci achieved the highest level of respondents’ willingness to reshare a call to social distancing, whereas celebrity spokesperson Kim Kardashian was least effective. Celebrity spokesperson Tom Hanks, the Government, and the no-spokesperson conditions took a middle ground. The likelihood of message resharing increased with age and when respondents expressed positive sentiments towards the spokesperson.

Subsequently, using generalized linear mixed models (GLMMs), we performed linear regression to test if the likelihood of message sharing varies by spokesperson, and whether this variation might be dependent on the respondents’ country and age (parametrized in terms of three statistically derived age groups, see S2 Fig and S2 Table in S1 File). These effects were tested in a single model while controlling for all other demographic and attitudinal measures (see *Materials and methods*). Our analysis revealed significant main effects for spokesperson (*p* < 0.001, *d* = 0.45), country (*p* < 0.001, *d* = 0.48), and age group (*p* < 0.001, *d* = 0.14) on message sharing (Fig 3A and 3B). S3 and S4 Tables in S1 File provide model details and all pairwise comparisons and effect sizes.

Out of all spokesperson conditions, Fauci had the greatest effect on message sharing (Fig 3A and 3B). Specifically, on the 7-point Likert scale, Fauci’s effect was on average greater (all *p* < 0.05) by 0.28 points relative to the no-spokesperson condition (95% CI = [0.10, 0.47], Cohen’s *d* = 0.07 standard deviations), by 0.51 points relative to the elected government official (95% CI = [0.30, 0.72], *d* = 0.12), by 0.67 points relative to Hanks (95% CI = [0.46, 0.89], *d* = 0.17), and by 1.85 points relative to Kardashian (95% CI = [1.63, 2.07], *d* = 0.45). Moreover, message sharing of respondents in the no-spokesperson condition was significantly higher (all *p* < 0.05) than those in the Government (mean difference [MD] = 0.23 points, 95% CI = [0.04, 0.42], *d* = 0.05), Hanks (MD = 0.39 points, 95% CI = [0.19, 0.59], *d* = 0.10), and Kardashian (MD = 1.57 points, 95% CI = [1.34, 1.80], *d* = 0.38) conditions. Message sharing of respondents in the Government condition was on par with those in the Hanks condition (MD = 0.16 points, 95% CI = [-0.003, 0.33], *d* = 0.04, *p* > 0.05), but was significantly higher than those in the Kardashian condition by 1.34 points (95% CI = [1.34, 1.80], *d* = 0.31, *p* < 0.05). Finally, message sharing in the Hanks condition was higher than in the Kardashian condition by 1.18 points (95% CI = [0.96, 1.39], *d* = 0.28, *p* < 0.05). Taken together, these results show that Dr. Fauci achieved the highest level of the respondents’ willingness to reshare a call to social distancing, the celebrity spokesperson Kim Kardashian achieved the lowest level, and the elected government official, the celebrity spokesperson Tom Hanks, and the no-spokesperson condition took a middle ground.

Among all countries, Brazil had the highest likelihood of message sharing (Fig 3A and 3B), ranging from 0.10 standard deviations above South Korea to 0.40 standard deviations above Switzerland (all *p* < 0.05). As for age (Fig 3A and 3B), older respondents significantly indicated a higher likelihood of message sharing (all *p* < 0.05): old > young (*d* = 0.14); old > mid-age (*d* = 0.06); and mid-age > young (*d* = 0.08).

**Fig 4:**
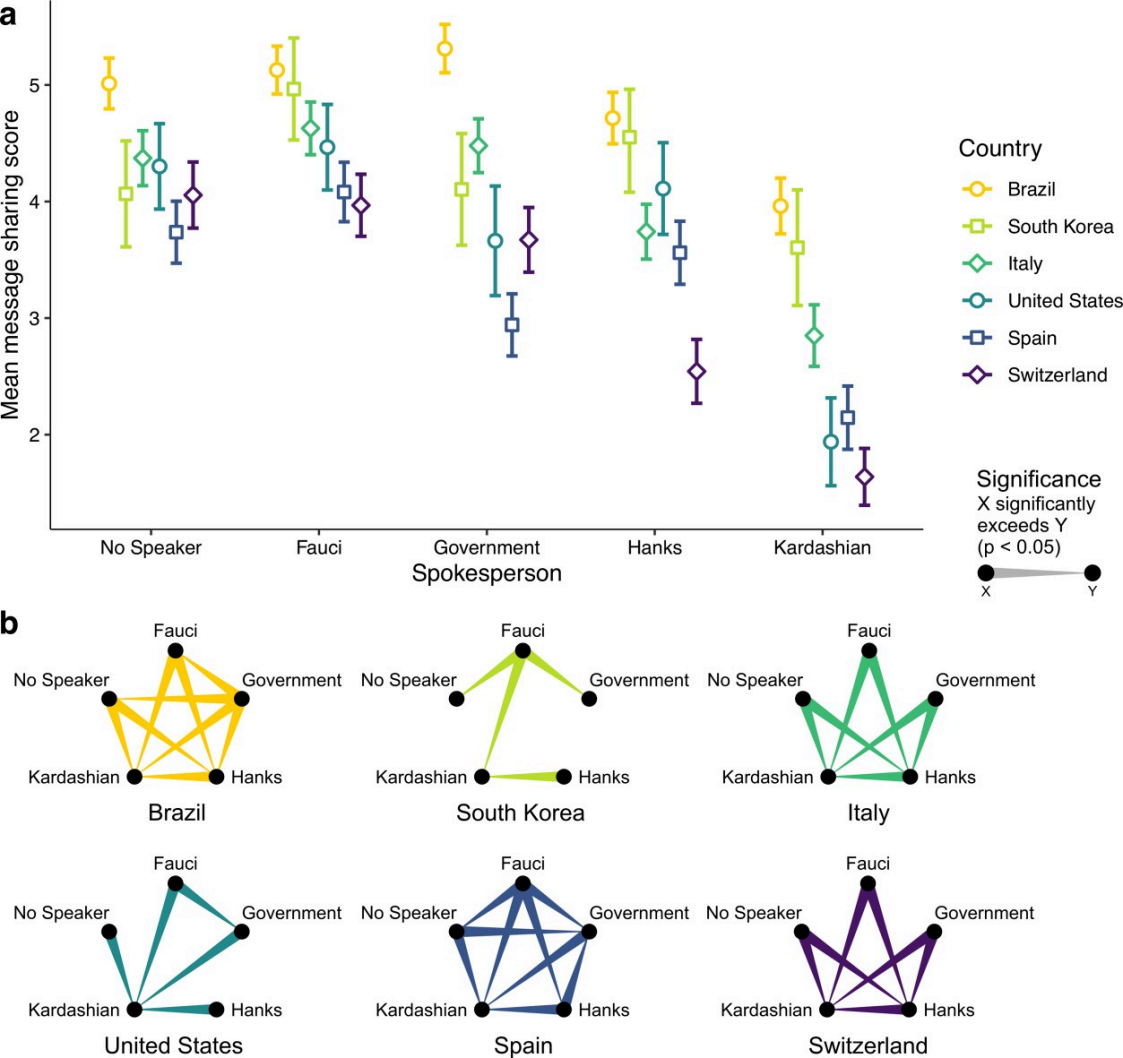
Country-by-spokesperson interaction on message sharing. **(a)** Message sharing score on a 1–7 Likert scale. Error bars represent 95% CIs. **(b)** Corresponding, color-coded significant pairwise comparisons, accounting for multiple comparisons via sequential Bonferroni correction. Only significant comparisons are shown.

The spokesperson effect on message sharing was moderated by country (*p* < 0.001, *d* = 0.26, Fig 4A and 4B). The government official was most effective in Brazil (*M* = 5.31, SE = 0.11, 95% CI = [5.11, 5.52]) and least effective in Spain (*M* = 2.94, SE = 0.14, 95% CI = [2.68, 3.21]). Fauci was significantly more effective than the elected government official in South Korea (*d* = 0.05), Spain (*d* = 0.14), and the United States (*d* = 0.14), and on par with the government in Italy and Switzerland. Celebrities were generally least effective. S5 Table in S1 File provides all pairwise comparisons and effect sizes, and S4 Fig in S1 File shows the frequency plots of message sharing by country and spokesperson.

**Fig 5:**
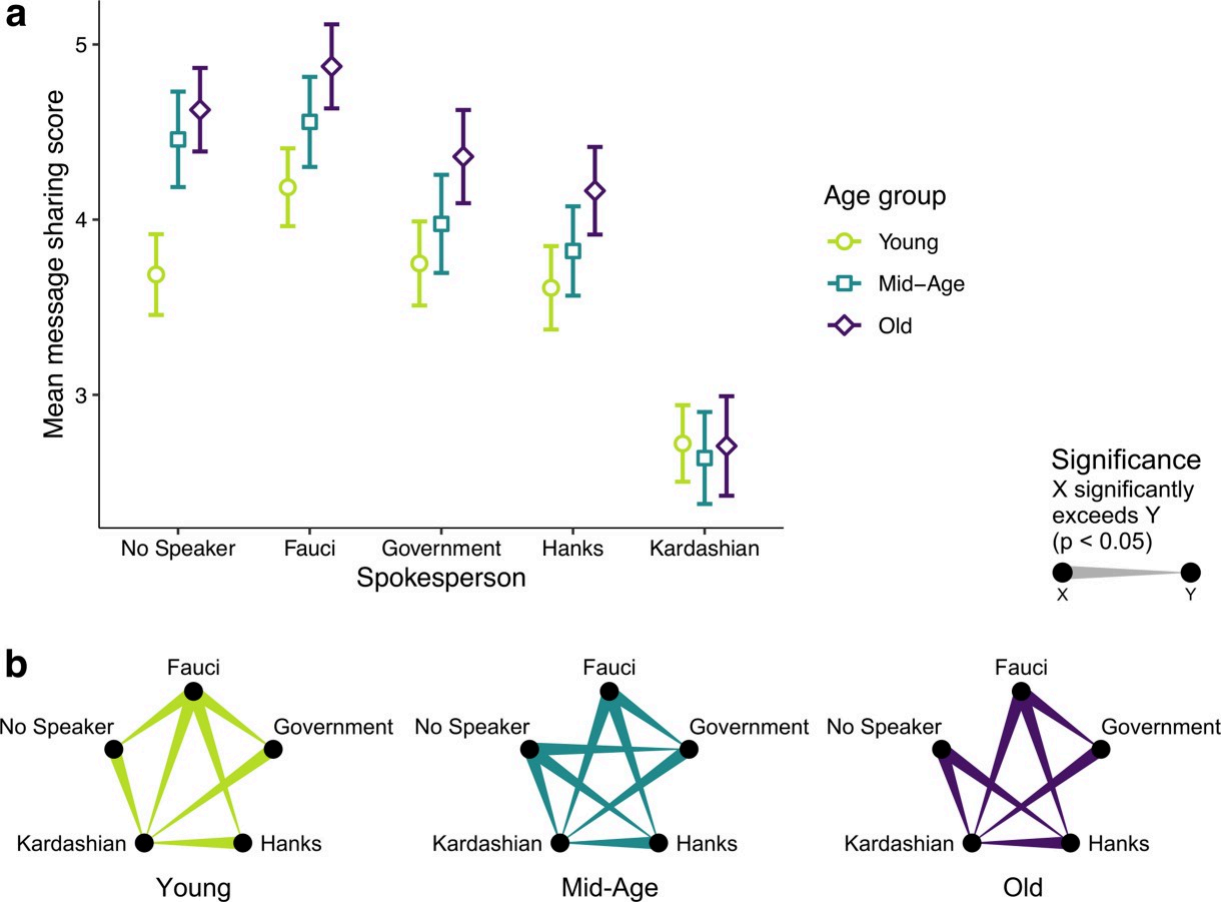
Spokesperson-by-age-group interaction on message sharing. **(a)** Message sharing score on a 1–7 Likert scale. Error bars represent 95% CIs. **(b)** Corresponding, color-coded significant pairwise comparisons, accounting for multiple comparisons via sequential Bonferroni correction. Only significant comparisons are shown.

In addition, the effect of the spokesperson condition on message sharing was moderated by age group (*p* < 0.001, *d* = 0.11, Fig 5A and 5B). Fauci was significantly more effective than all other spokespersons across all age groups (*d* between 0.06 and 0.26), and on par with the no-spokesperson condition among the mid- and old-age groups. S6 Table in S1 File provides all pairwise comparison results and effect sizes, and S5 Fig in S1 File shows the frequency plots of message sharing by spokesperson and age group.

Finally, the 3-way interaction of spokesperson, country, and age group on message sharing, although significant (*p* < 0.001, *d* = 0.18), did not reveal important deviations from the observations made from the 2-way interactions described above. S6 Fig in S1 File visualizes the 3-way interaction, S7 Table in S1 File provides all pairwise comparison results and effect sizes, and S7 Fig in S1 File shows the frequency plots of message sharing by country and age group.

**Fig 6:**
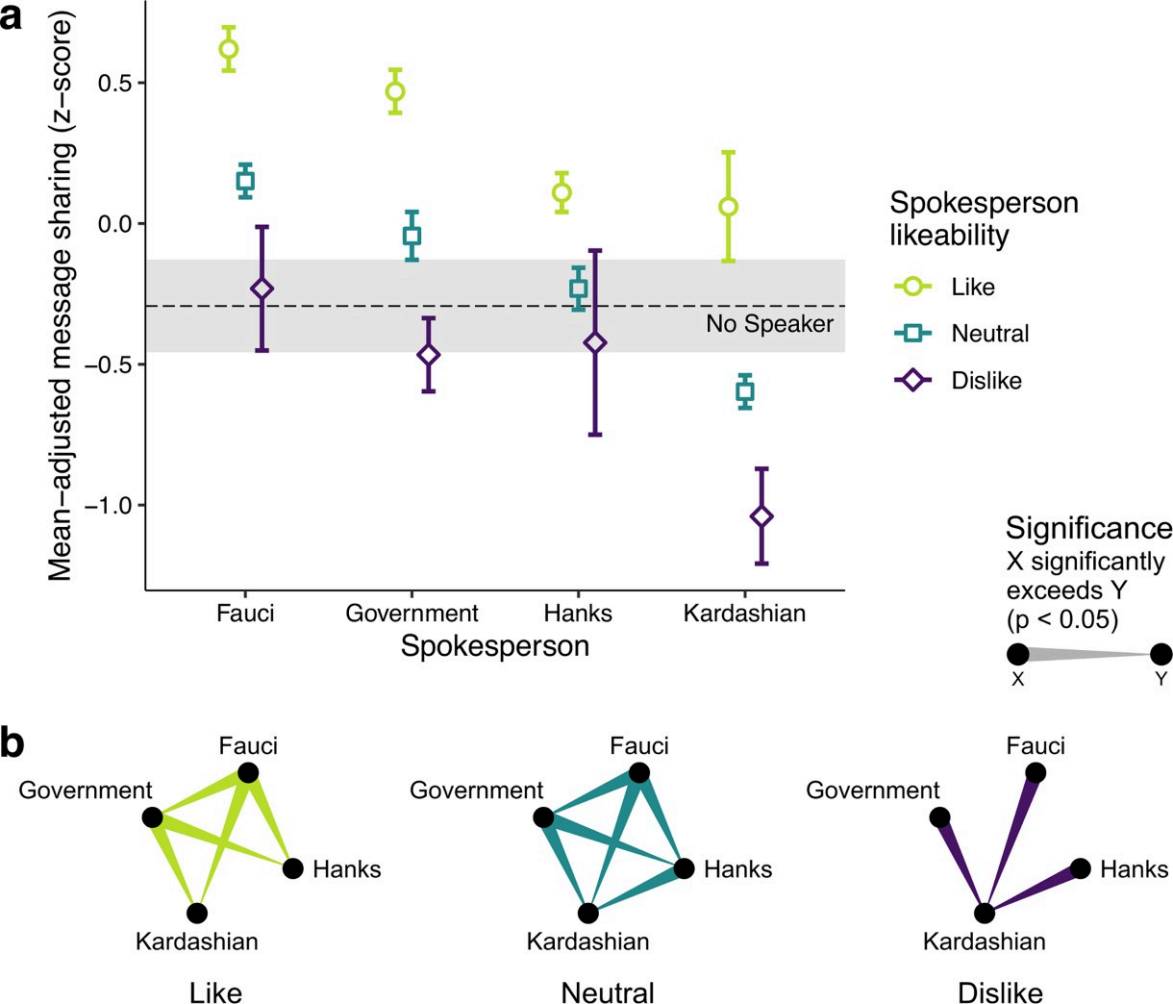
Spokesperson-by-likeability interaction on message sharing. **(a)** Message sharing score as the standardized residual of the message sharing scores (on a 1–7 Likert scale), adjusted for all demographic and attitudinal measures (see *Materials and methods*: *Statistical analyses*). Error bars represent 95% CIs. The dashed black line (95% CI, gray band) represents the effect for the no-spokesperson condition, for which a likeability could not be elicited. **(b)** Corresponding, color-coded significant pairwise comparisons, accounting for multiple comparisons via sequential Bonferroni correction. Only significant comparisons are shown.

Evidence suggests that celebrities who are viewed favorably consistently have positive effects on people’s opinions, attitudes, and behaviors [18, 19]. Thus, in a separate GLMM, we estimated in a linear regression the extent to which respondents’ sentiment towards the spokesperson affected the likelihood of message sharing. Being liked boosted the effect on message sharing for all spokespersons (*p* < 0.05, *d* = 0.07, S8 Table in S1 File), and particularly for social media personality Kardashian (Fig 6A and 6B). All effects among respondents who liked the spokespersons were significantly higher than the effect of the no-spokesperson condition (note the non-overlapping confidence intervals, Fig 6A). Notably, Fauci retained his status as the most influential spokesperson on message sharing across all likeability levels, namely among those who expressed positive (*d* = 0.06 to *d* = 0.20), neutral (*d* = 0.08 to *d* = 0.36), and even negative (*d* = 0.02 to *d* = 0.12) sentiments towards the spokespersons, although for the latter his effect was only significantly greater than for Kardashian. S9 Table in S1 File provides the pairwise comparisons and effect sizes for the interaction of spokesperson and likeability. The effect of likeability was evident for respondents in all countries (*p* < 0.001, *d* = 0.13, S8 Fig and S8, S10 Tables in S1 File).

## Discussion

Overall, the results revealed large differences between the four spokespersons in terms of their effectiveness as advocates for social distancing. Considering that, in the context of a pandemic, even small effects can translate into saving many lives [35], this constitutes a consequential result. Across demographic strata, the immunology expert Dr. Anthony Fauci achieved the highest level of willingness to reshare a call to social distancing, followed by the elected government official and celebrity actor Tom Hanks. Media personality Kim Kardashian was by far the least effective spokesperson for social distancing, across age groups and countries. Remarkably, while the magnitude of the effect increased for all spokespersons among respondents who expressed favorable sentiments towards them, their relative effect on message sharing was retained (expert > government > celebrities) and persisted across national and cultural boundaries.

Thus, empowering experts during the pandemic could not be more important, particularly when misinformation by high-profile figures can have fatal consequences during the pandemic [36], and especially in its early stages [37]. Even in the presence of a preventive vaccine and effective treatment, efforts to mitigate the outbreak will necessarily continue to rely on abiding by social and physical distancing rules, which may need to be sustained as late as 2022 [4]. Consequently, enlisting and supporting the most effective spokespersons for public health messaging will be critical in slowing transmission and mobilizing large-scale social distancing interventions. This was recognized as a key factor in the handling of the 2003 SARS outbreak in Toronto [5] and the 2009 H1N1 influenza pandemic [38]. In order to counter misinformation and the undermining of expert advice [36, 37], expert impact can be bolstered if sanctioned by governments, and similarly governments can increase their effectiveness by basing their decisions on the most up-to-date scientific advice and evidence, particularly when decisions need to be made under the uncertain conditions of a pandemic [38].

The results of this study should be considered in the light of its limitations. For example, our study did not measure actual message sharing, but respondents’ stated willingness to do so. Evidence suggests, however, that self-reports of intended behavior during the COVID-19 pandemic do in fact reflect real behavior [39] and self-reported intent to share content on social media has been linked to subsequent sharing behavior [24]. Moreover, we tested the effectiveness of four spokespersons only. Future research should extend the study to other spokespersons from different social spheres, such as leaders within the faith sector. Indeed, our data show that religiosity is one of the highest correlates of message sharing (*r* = 0.23, *p* = 3.21x10^-146^, Fig 2), and research shows that enlisting religious leaders during the West African Ebola crisis proved critical in slowing transmission through the revision of safe burial practices [40]. Given the sudden, worldwide spread of COVID-19 outside of China in March 2020, one might also argue that Dr. Fauci simply filled a vacuum of trust at the time of the study. While this is almost certainly the case (for some demographics) in the United States, he is by no means an uncontroversial figure. It is also questionable if his rise to prominence can be considered a global phenomenon, yet our findings are consistent across all countries in our study, including those with notably different cultural background, in which Dr. Fauci is likely to be considered “just” an expert (with the possible exception of Brazil, where the minister of health filled a similar opposition role to the government as Dr. Fauci did in the United States). We emphasize that, although the present results clearly show Fauci’s prominent role, they do not allow us to draw conclusions regarding the underlying causal mechanisms. To elucidate what specific properties (being a proven expert, holding an MD, being old, being visible on TV, etc.) make some spokespersons more effective than others, future work should repeat our study with a range of nearly-identical, fabricated personas that differ only in carefully selected demographic and biographic attributes.

We also observed a heterogeneous spokesperson effect on message sharing across different segments of the population (Fig 5A). This suggests that multiple spokespersons might be needed to achieve equal effects across the population, a strategy that is also supported by research on social contagion, which suggests that message resharing is likely to increase if encouraged by multiple non-overlapping social circles [41]. Similarly, it is important to remember that the observed effects are merely the result of a single message. The effect of multiple messages from a single spokesperson would be intriguing for future research to explore. With regard to spokesperson likeability, it is worth noting that we elicited spokesperson likeability after the treatment and thus cannot rule out any effects that this stimulus may have had on the likeability ratings. Furthermore, it is conceivable that the spokesperson identity may have influenced participants’ decisions to complete the survey or refrain from submitting it. Future studies could address this reverse effect of the stimulus on spokesperson likeability. Such an effect may be further compounded by partisan bias, which has, for example, shaped the reception of, and adherence to, health measures in the United States [42], indicating that the respondent’s ideology may play a role in the effectiveness of a spokesperson to successfully deliver the message. Furthermore, it seems likely that the success of promoting specific message content (e.g., social distancing, vaccination, or the use of a tracing app, for example) may differ for different spokespersons. Finally, as our findings cannot yet speak to long-term effects, future research should replicate these results at different stages of the pandemic to determine if different spokespersons are most effective at different stages of the pandemic.

Our study contributes to the development of evidence-based knowledge regarding the effectiveness of prominent official and non-official public figures in communicating public health messaging during the COVD-19 pandemic. The findings presented here can help governments shape effective strategies for communicating behaviors aimed at mitigating the COVID-19 pandemic, including prospective challenges associated with vaccination and proximity-tracing compliance. Numerous celebrities are advocating for social distancing and, maybe partly in response to a general decline in experts’ credibility as perceived by the public [43], governments have started to enlist celebrities as spokespersons [44]. While it is possible that celebrities can bring heightened awareness to health issues [20], especially among their fan base (Fig 6A), this awareness may not be associated with heightened public understanding of related risks and treatment [45]. Our findings thus serve as a reminder to governments and experts not to underestimate their own power to inform and persuade.

## Supporting information

S1 File (PDF)

## References

[1] Betsch C. How behavioural science data helps mitigate the COVID-19 crisis. Nat Hum Behav. 2020 10.1038/s41562-020-0866-1 32221514 PMC7101898

[2] Wilder-Smith A, Freedman DO. Isolation, quarantine, social distancing and community containment: pivotal role for old-style public health measures in the novel coronavirus (2019-nCoV) outbreak. J Travel Med. 2020 10.1093/jtm/taaa020 32052841 PMC7107565

[3] Buckee CO, Balsari S, Chan J, Crosas M, Dominici F, Gasser U, et al Aggregated mobility data could help fight COVID-19. Science. 2020 10.1126/science.abb8021 32205458

[4] Kissler SM, Tedijanto C, Goldstein E, Grad YH, Lipsitch M, Projecting the transmission dynamics of SARS-CoV-2 through the postpandemic period. Science. 2020 10.1126/science.abb5793 32291278 PMC7164482

[5] DiGiovanni C, Conley J, Chiu D, Zaborski J. Factors influencing compliance with quarantine in Toronto during the 2003 SARS outbreak. Biosecur Bioterror. 2004 10.1089/bsp.2004.2.265 15650436

[6] Jamison DT, Summers LH, Alleyne G, Arrow KJ, Berkley S, Binagwaho A, et al Global health 2035: a world converging within a generation. Lancet. 2013 10.1016/S0140-6736(13)62105-4 24309475

[7] Van Bavel JJ, Baicker K, Boggio PS, Capraro V, Cichocka A, Cikara M, et al Using social and behavioural science to support COVID-19 pandemic response. Nat Hum Behav. 2020 10.1038/s41562-020-0884-z 32355299

[8] O’Keefe DJ, Persuasion: Theory and Research. 2nd edition Sage; 2002

[9] Rimer BK, Kreuter MW. Advancing tailored health communication: A persuasion and message effects perspective. J Commun. 2006 10.1111/j.1460-2466.2006.00289.x

[10] Sutton J, Gibson CB, Phillips NE, Spiro ES, League C, Johnson B, et al A cross-hazard analysis of terse message retransmission on Twitter. Proc Natl Acad Sci. 2015 10.1073/pnas.1508916112 26627233 PMC4672824

[11] O'Keefe DJ, Jensen JD. Do loss-framed persuasive messages engender greater message processing than do gain-framed messages? A meta-analytic review. Commun. Stud. 2008 10.1080/10510970701849388

[12] Feinberg M, Willer R. Moral reframing: A technique for effective and persuasive communication across political divides. Soc Personal Psychol Compass. 2019 10.1111/spc3.12501

[13] Matz SC, Kosinski M, Nave G, Stillwell DJ, Psychological targeting as an effective approach to digital mass persuasion. Proc Natl Acad Sci. 2017 10.1073/pnas.1710966114 29133409 PMC5715760

[14] Seeger MW. Best Practices in Crisis Communication: An Expert Panel Process, J Appl Commun Res. 2006 10.1080/00909880600769944

[15] Lyu SY, Chen RY, Wang SS, Weng YL, Peng EYC, Lee MB. Perception of Spokespersons' Performance and Characteristics in Crisis Communication: Experience of the 2003 Severe Acute Respiratory Syndrome Outbreak in Taiwan. J Formos Med Assoc. 2013 10.1016/j.jfma.2012.12.005 24120151 PMC7135798

[16] Austin JL. How to Do Things with Words. Oxford University Press; 1962

[17] Sutton J, Spiro ES, Johnson B, Fitzhugh S, Gibson B, Butts CT. Warning tweets: serial transmission of messages during the warning phase of a disaster event. Inf. Commun. Soc. 2014 10.1080/1369118X.2013.862561

[18] Jackson DJ. The effects of celebrity endorsements of ideas and presidential candidates. *J Political Mark*. 2018 10.1080/15377857.2018.1501530

[19] Jackson D, Darrow T. The influence of celebrity endorsements on young adults’ political opinions. Int J Press/Politics. 2005 10.1177/1081180X05279278

[20] Beck CS, Aubuchon SM, McKenna TP, Ruhl S, Simmons N. Blurring personal health and public priorities: An analysis of celebrity health narratives in the public sphere. Health Commun. 2014 10.1080/10410236.2012.741668 23548050

[21] Frizzell C. Public opinion and foreign policy: the effects of celebrity endorsements. Soc Sci J. 2011 10.1016/j.soscij.2010.07.009 21448248 PMC3063942

[22] Gaines BJ. Where's the rally? Approval and trust of the president, cabinet, congress, and government since September 11. PS Political Sci Politics. 2002 10.1017/S1049096502000793

[23] Boin A, 't Hart P, Stern E, Sundelius B. The *Politics of Crisis Management*: *Public Leadership Under Pressure*. Cambridge University Press; 2016 10.1017/CBO9780511490880

[24] Mosleh M, Pennycook G, Rand D, Self-reported Willingness to Share Political News Articles in Online Surveys Correlates with Actual Sharing on Twitter. PLoS One. 2020 10.1371/journal.pone.0228882 32040539 PMC7010247

[25] Zhang J, Litvinova M, Liang Y, Wang Y, Wang W, Zhao S, et al Changes in contact patterns shape the dynamics of the COVID-19 outbreak in China. Science. 2020 10.1126/science.abb8001 32350060 PMC7199529

[26] Smith CT, De Houwer J. The Impact of Persuasive Messages on IAT Performance is Moderated by Source Attractiveness and Likeability. Soc Psychol. 2014 10.1027/1864-9335/a000208

[27] Roskos-Ewoldsen DR, Fazio RH. The Accessibility of Source Likability as a Determinant of Persuasion. Pers Soc Psychol Bull. 1992 10.1177/0146167292181004 1403611

[28] Johns Hopkins University. COVID-19 information and resources for JHU [cited March 20, 2020]. Available from: https://hub.jhu.edu/novel-coronavirus-information

[29] WHO. WHO Director-General's opening remarks at the media briefing on COVID-19–11 March 2020 [cited May 19, 2020]. Available from: https://www.who.int/dg/speeches/detail/who-director-general-s-opening-remarks-at-the-media-briefing-on-covid-19—11-march-2020

[30] Andrews FM, Herzog AR. The quality of survey data as related to age of respondent. J Am Stat Assoc. 1986 10.1080/01621459.1986.10478284

[31] Rousseeuw PJ. Silhouettes: a graphical aid to the interpretation and validation of cluster analysis. J Comput Appl Math. 1987 10.1016/0377-0427(87)90125-7

[32] Bish A, Michie S. Demographic and attitudinal determinants of protective behaviours during a pandemic: A review. Br J Health Psychol. 2010 10.1348/135910710X485826 20109274 PMC7185452

[33] Pfattheicher S, Nockur L, Böhm R, Sassenrath C, Petersen MB. The Emotional Path to Action: Empathy Promotes Physical Distancing and Wearing of Face Masks During the COVID-19 Pandemic. Psychol Sci. 2020 10.1177/0956797620964422 32993455

[34] Wise T, Zbozinek TD, Michelini G, Hagan CC, Mobbs D. Changes in risk perception and self-reported protective behaviour during the first week of the COVID-19 pandemic in the United States. R Soc Open Sci. 2020 10.1098/rsos.200742 33047037 PMC7540790

[35] Strong K, Mathers C, Leeder S, Beaglehole R. Preventing chronic diseases: how many lives can we save? Lancet. 2005 10.1016/S0140-6736(05)67341-2 16257345

[36] Liu M, Caputi TL, Dredze M, Kesselheim AS, Ayers JW. Internet Searches for Unproven COVID-19 Therapies in the United States. JAMA Intern Med. 2020 10.1001/jamainternmed.2020.1764 32347895 PMC7191468

[37] Bursztyn L, Rao A, Roth C, Yanagizawa-Drott D, Misinformation During a Pandemic. [Working Paper]. 2020. Available from 10.2139/ssrn.3580487

[38] Fineberg HV. Pandemic preparedness and response—lessons from the H1N1 influenza of 2009. N Engl J Med. 2014 10.1056/NEJMra1208802 24693893

[39] Gollwitzer A, Martel C, Marshall J, Höhs JM, Bargh JA. Connecting Self-Reported Social Distancing to Real-World Behavior at the Individual and U.S. State Level. [Preprint]. 2020 Available from https://psyarxiv.com/kvnwp/

[40] Greyling C, Maulit JA, Parry S, Robinson D, Smith S, Street A, et al Lessons from the Faith-Driven Response to the West Africa Ebola Epidemic. Rev Faith Int Aff. 2016 10.1080/15570274.2016.1215829

[41] Ugander J, Backstrom L, Marlow C, Kleinberg J. Structural diversity in social contagion. Proc Natl Acad Sci. 2012 10.1073/pnas.1116502109 22474360 PMC3341012

[42] Gollust SE, Nagler RH, Franklin Fowler E. The Emergence of COVID-19 in the U.S.: A Public Health and Political Communication Crisis. J Health Polit Policy Law. 2020 10.1215/03616878-8641506 32464658

[43] Bucchi M. Facing the challenges of science communication 2.0: quality, credibility and expertise. EFSA J. 2019 10.2903/j.efsa.2019.e170702 32626439 PMC7015522

[44] Berset A. Announcement of the Swiss Federal Office of Public Health (BAG) [cited March 22, 2020]. Available from: https://www.instagram.com/p/B996tSCBjBQ

[45] Borzekowski DL, Guan Y, Smith KC, Erby LH, Roter DL, The Angelina effect: immediate reach, grasp, and impact of going public. Genet Med. 2014 10.1038/gim.2013.181 24357847

