# Supplementary material for: The effect of spokesperson attribution on public health message sharing during the COVID-19 pandemic
Source: PLoS One. 2021 Feb 3;16(2):e0245100. doi: 10.1371/journal.pone.0245100 (PMC7857592; doi:10.1371/journal.pone.0245100)

The Effect of Spokesperson Attribution on Public Health Message Sharing During the COVID-19 Pandemic

Ahmad Abu-Akel, Andreas Spitz, Robert West

This PDF file includes:

- Supplementary Text
- Figures S1 to S8
- Tables S1 to S10

Other Supplementary Materials for this manuscript include the following:

- Appendix A: Survey
- Appendix B: Web sources of official announcements and support statements
- Appendix C: Timeline of government issuance of nine social distancing measures
- Appendix D: Facebook advertisement

Supplementary Text

Table S1. Sample characteristics for the overall sample and by Country[1] (N = 12,194)

Variable	Overall	BR	CH	ES	IT	KR	US
Age[2]	37.04 (14.80)	37.7 (14.66)	38.54 (14.53)	36.4 (14.65)	44.97 (17.08)	41.19 (17.40)	34.98 (13.84)
**Gender**							
Female	7316	2587	899	1320	1841	246	423
Male	4878	1755	766	812	1080	229	236
**Employment**							
Student	1807	455	359	289	578	68	58
Employed	5547	1920	983	977	1181	147	339
Self-employed	1731	975	105	173	333	96	49
Unemployed	2180	738	82	519	641	98	102
Retired	929	254	136	174	188	66	111
**Education years**							
No schooling	235	93	5	44	17	33	43
1-6 years	369	131	42	66	37	14	79
7-13 years	2670	862	322	360	879	95	152
14-16 years	3442	1166	468	552	903	151	202
17-18 years	2269	834	357	388	549	55	86
Over 18 years	3209	1256	471	722	536	127	97
**Settlement Size**							
Village	1601	178	425	366	540	17	75
Small Town	2592	598	505	487	812	31	159
Town	3348	1224	330	579	955	66	194
City	2812	1260	377	489	397	132	157
Metropolitan	1841	1082	28	211	217	229	74
Household Size[2]	3.14 (1.42)	3.37 (1.54)	2.82 (1.41)	3.05 (1.23)	3.11 (1.30)	2.94 (1.24)	3.08 (1.67)
Likelihood of Message Sharing[1]	4.42 (2.40)	5.28 (2.17)	3.17 (2.27)	3.82 (2.41)	4.31 (2.32)	4.45 (2.32)	4.28 (2.45)
Support of S/PD[2,3]	6.37 (1.21)	6.33 (1.28)	6.58 (0.90)	6.60 (0.97)	6.16 (1.34)	5.96 (1.43)	6.55 (1.04)
Current Practice of S/PD measures[2,3]	6.24 (1.10)	5.94 (1.27)	6.29 (0.93)	6.50 (0.89)	6.54 (0.84)	5.79 (1.29)	6.19 (1.09)
Intention to practice S/PD in the future[2,3]	6.13 (1.24)	5.84 (1.48)	6.20 (0.98)	6.50 (0.90)	6.30 (1.08)	5.67 (1.42)	6.25 (1.10)
Number of supported S/PD[1,2] measures	6.70 (2.37)	6.50 (2.49)	6.61 (1.86)	7.55 (2.05)	6.60 (2.45)	4.82 (2.19)	7.23 (2.18)
Concern Situation[2]	5.81 (1.45)	6.04 (1.44)	4.93 (1.45)	6.06 (1.27)	5.80 (1.35)	5.67 (1.62)	5.89 (1.43)
Concern Others[2]	6.14 (1.28)	6.41 (1.15)	5.49 (1.35)	6.37 (1.09)	5.99 (1.35)	5.68 (1.49)	6.28 (1.20)
Others' Practice S/PD[2,3]	4.12 (1.27)	3.72 (1.29)	4.63 (1.02)	4.41 (1.22)	4.30 (1.17)	4.28 (1.36)	3.67 (1.29)
Liberty of Movement[2]	1.85 (1.58)	2.09 (1.74)	1.90 (1.39)	1.49 (1.38)	1.33 (1.05)	3.14 (1.84)	2.73 (2.01)
Satisfaction from Government[2]	3.84 (2.06)	3.37 (2.09)	4.73 (1.71)	3.20 (2.01)	4.52 (1.78)	4.68 (2.22)	3.22 (2.03)
Government prioritizes Public Health over Economy[2]	3.36 (2.08)	2.37 (1.83)	4.19 (1.80)	3.30 (2.09)	4.32 (1.85)	4.32 (2.11)	3.08 (2.00)
Religiosity[2]	3.79 (2.36)	5.04 (2.20)	2.49 (1.91)	2.74 (2.04)	3.39 (2.14)	3.70 (2.32)	4.10 (2.36)

Variable	Overall	BR	CH	ES	IT	KR	US
**General Health**							
Very bad	132	51	7	27	37	1	9
Bad	733	211	66	235	144	23	54
Average	3235	1055	266	779	798	156	181
Good	4972	1912	751	681	1220	169	239
Very good	3122	1113	575	410	722	126	176
**Infection %**							
0-9%	5576	2510	635	781	1064	310	276
10-19%	2125	702	399	374	450	68	132
20-29%	1336	377	242	281	313	34	89
30-39%	1082	301	162	224	310	21	64
40-49%	621	175	80	129	193	14	30
50-59%	529	116	86	107	178	8	34
60-69%	369	65	29	102	165	2	6
70-79%	292	52	25	67	125	7	16
80-89%	170	31	2	44	78	8	7
90-100%	94	13	5	23	45	3	5
**Awareness of S/PD**							
Yes	11872	4310	1624	2089	2735	463	651
No	322	32	41	43	186	12	8
**Attitude to speaker**							
No Speaker[4]	2401	844	319	415	562	118	143
Government (*N*=2,612)							
Like	1010	364	147	68	346	40	45
Neutral	1157	373	196	181	226	35	46
Dislike	445	41	22	203	106	25	48
Fauci (*N* = 2293)							
Like	371	158	40	56	56	4	57
Neutral	1912	622	296	362	494	76	62
Dislike	10	7	0	1	2	0	0
Hanks (*N*=2535)							
Like	1173	495	134	152	275	31	86
Neutral	1332	467	184	262	310	66	43
Dislike	30	10	5	7	5	1	2
Kim (*N*= 2,353)							
Like	162	93	8	20	30	3	8
Neutral	1947	733	243	339	479	72	81
Dislike	244	35	71	66	30	4	38

[1] Country Key: BR = Brazil; CH = Switzerland; ES = Spain; IT = Italy; KR = South Korea; US = United States. [2] Mean (SD); [3] S/PD = Social/Physical Distancing; [4] Likeability rating could not be solicited in the 'No Speaker' condition.

Table S2. Demographic overview of the three age groups by country and for the overall sample

**Country**[1]	**BR**	**CH**	**ES**	**IT**	**KR**	**US**	**Overall**
**N**							
Young	2358	786	868	1492	149	278	5931
Mid-Age	1253	535	745	814	111	160	3618
Old	731	344	519	615	215	221	2645
Gender (M/F)							
Young	925/1433	373/413	278/590	567/925	57/92	101/177	2301/3630
Mid-Age	518/735	236/299	324/421	302/512	51/60	59/101	1490/2128
Old	312/419	157/187	210/309	211/404	121/94	76/145	1087/1558
Age (Mean & SD)							
Young	24.59 (4.09)	25.28 (3.61)	24.70 (3.51)	24.57 (3.71)	24.03 (4.14)	24.40 (3.92)	24.67 (3.85)
Mid-Age	40.13 (4.99)	40.95 (5.15)	39.87 (4.87)	40.31 (5.13)	41.47 (5.23)	40.69 (5.46)	40.30 (5.06)
Old	59.69 (6.06)	61.00 (7.52)	59.78 (6.19)	59.91 (6.27)	61.28 (6.42)	62.67 (6.75)	60.31 (6.49)

[1] Country Key: BR = Brazil; CH = Switzerland; ES = Spain; IT = Italy; KR = South Korea; US = United States

Table S3. Model summary of message sharing

Source	F	df1	df2	p-value[1]	η_p^2	Cohen's d
Corrected Model	58.931	163	12030	**0.000**	0.444	1.79
Spokesperson	148.899	4	12030	**0.000**	0.047	0.45
Country	136.611	5	12030	**0.000**	0.054	0.48
Age Group	31.253	2	12030	**0.000**	0.005	0.14
Spokesperson x Country	10.664	20	12030	**0.000**	0.017	0.27
Spokesperson x Age Group	4.372	8	12030	**0.000**	0.003	0.11
Spokesperson x Country x Age Group	1.949	50	12030	**0.000**	0.008	0.18
Gender	0.345	1	12030	0.557	0.000	0.01
Employment	0.945	1	12030	0.437	0.000	0.02
Education	3.481	5	12030	**0.004**	0.001	0.08
Household size	0.328	1	12030	0.567	0.000	0.01
City size	0.734	4	12030	0.569	0.000	0.03
Concern for the situation	28.590	6	12030	**0.000**	0.014	0.24
Concern for others	16.712	6	12030	**0.000**	0.008	0.18
Others' practice of SD	0.815	6	12030	0.558	0.000	0.04
Freedom of movement	0.998	6	12030	0.424	0.000	0.04
Infection percent	2.282	9	12030	**0.015**	0.002	0.08
Subjective Health	0.578	4	12030	0.678	0.000	0.03
Satisfaction from government	4.801	6	12030	**0.000**	0.002	0.10
Public health over Economy	4.468	6	12030	**0.000**	0.002	0.09
Religiosity	11.613	6	12030	**0.000**	0.006	0.15
Support of SD	271.469	1	12030	**0.000**	0.022	0.30
Current practice of SD	2.254	1	12030	0.134	0.000	0.03
Future practice of SD	35.782	1	12030	**0.000**	0.003	0.11
Number of SD measures endorsed	41.224	1	12030	**0.000**	0.003	0.12

[1] p-values < 0.05 are highlighted in bold

Table S4. Pairwise comparisons of main effects (Spokesperson, Country, Age group) and effect sizes

Spokesperson Contrast	MD	SE	t	df	Adj. P	LCI95	UCI95	Cohen's d
Fauci - No speaker	0.282	0.078	3.624	12030	0.000	0.096	0.469	0.07
Fauci - Government	0.511	0.083	6.188	12030	0.001	0.298	0.723	0.12
Fauci - Hanks	0.674	0.079	8.581	12030	0.000	0.459	0.889	0.17
Fauci - Kardashian	1.850	0.080	22.987	12030	0.000	1.633	2.066	0.45
Government - No speaker	-0.229	0.084	-2.713	12030	0.013	-0.418	-0.040	-0.05
Government - Hanks	0.163	0.085	1.924	12030	0.054	-0.003	0.329	0.04
Government - Kardashian	1.339	0.086	15.492	12030	0.000	1.099	1.579	0.31
No speaker - Hanks	0.392	0.080	4.882	12030	0.000	0.191	0.592	0.10
No speaker - Kardashian	1.568	0.082	19.097	12030	0.000	1.337	1.798	0.38
Hanks - Kardashian	1.176	0.083	14.229	12030	0.000	0.958	1.394	0.28
**Country Contrast[1]**	**MD**	**SE**	**t**	**df**	**Adj. P**	**LCI95**	**UCI95**	**Cohen's d**
BR-CH	1.651	0.077	21.350	12030	0.000	1.424	1.878	0.39
BR-ES	1.533	0.070	22.040	12030	0.000	0.133	1.736	0.40
BR-IT	0.812	0.066	12.272	12030	0.000	0.621	1.004	0.22
BR-KR	0.574	0.110	5.233	12030	0.000	0.279	0.869	0.10
BR-US	1.130	0.095	11.937	12030	0.000	0.859	1.402	0.22
CH-ES	-0.118	0.076	-1.548	12030	0.122	-0.268	0.031	-0.03
CH-IT	-0.839	0.071	-11.876	12030	0.000	-1.039	-0.639	-0.22
CH-KR	-1.077	0.122	-8.853	12030	0.000	-1.418	-0.735	-0.16
CH-US	-0.521	0.106	-4.924	12030	0.000	-0.800	-0.242	-0.09
ES-IT	-0.720	0.067	-10.747	12030	0.000	-0.906	-0.535	-0.20
ES-KR	-0.959	0.120	-8.017	12030	0.000	-1.286	-0.632	-0.15
ES-US	-0.403	0.102	-3.943	12030	0.000	-0.658	-0.148	-0.07
IT-KR	-0.238	0.117	-2.033	12030	0.084	-0.501	0.024	-0.04
IT-US	0.318	0.101	3.153	12030	0.005	0.077	0.559	0.06
KR-US	0.556	0.136	4.096	12030	0.000	0.206	0.906	0.07
**Age Group Contrast**	**MD**	**SE**	**t**	**df**	**Adj. P**	**LCI95**	**UCI95**	**Cohen's d**
Old-Young	0.556	0.071	7.825	12030	0.000	0.386	0.726	0.14
Old- Mid age	0.257	0.072	3.548	12030	0.000	0.115	0.398	0.06
Mid age - Young	0.299	0.066	4.513	12030	0.000	0.151	0.448	0.08

[1] Country Key: BR = Brazil; CH = Switzerland; ES = Spain; IT = Italy; KR = South Korea; US = United States

Table S5. Pairwise comparisons of the spokesperson × country interaction

Country[1]	Comparison	MD	SE	t	df	Adj. P	LCI95	UCI95	Cohen's d
BR	Fauci - No speaker	0.115	0.087	1.322	12030	0.186	-0.055	0.285	0.02
	Fauci - Government	-0.185	0.081	-2.271	12030	0.046	-0.367	-0.002	-0.04
	Fauci - Hanks	0.412	0.089	4.662	12030	0.000	0.182	0.641	0.09
	Fauci - Kardashian	1.166	0.103	11.304	12030	0.000	0.884	1.448	0.21
	Government - No speaker	0.299	0.085	3.524	12030	0.002	0.087	0.512	0.06
	Government - Hanks	0.596	0.087	6.836	12030	0.000	0.366	0.827	0.12
	Government - Kardashian	1.350	0.102	13.282	12030	0.000	1.068	1.632	0.24
	No speaker - Hanks	0.297	0.093	3.208	12030	0.004	0.075	0.519	0.06
	No speaker - Kardashian	1.051	0.106	9.891	12030	0.000	0.753	1.349	0.18
	Hanks - Kardashian	0.754	0.108	6.979	12030	0.000	0.463	1.045	0.13
CH	Fauci - No speaker	-0.087	0.163	-0.536	12030	0.592	-0.406	0.232	-0.01
	Fauci - Government	0.297	0.160	1.849	12030	0.129	-0.063	0.656	0.03
	Fauci - Hanks	1.425	0.160	8.911	12030	0.000	0.995	1.855	0.16
	Fauci - Kardashian	2.330	0.145	16.105	12030	0.000	1.948	2.712	0.29
	Government - No speaker	-0.384	0.168	-2.289	12030	0.066	-0.785	0.018	-0.04
	Government - Hanks	1.129	0.165	6.841	12030	0.000	0.704	1.554	0.12
	Government - Kardashian	2.033	0.150	13.545	12030	0.000	1.623	2.444	0.25
	No speaker - Hanks	1.512	0.167	9.065	12030	0.000	1.044	1.981	0.17
	No speaker - Kardashian	2.417	0.152	15.855	12030	0.000	1.994	2.840	0.29
	Hanks - Kardashian	0.905	0.149	6.072	12030	0.000	0.533	1.277	0.11
ES	Fauci - No speaker	0.345	0.148	2.326	12030	0.040	0.013	0.677	0.04
	Fauci - Government	1.140	0.149	7.638	12030	0.000	0.739	1.542	0.14
	Fauci - Hanks	0.522	0.152	3.431	12030	0.002	0.158	0.886	0.06
	Fauci - Kardashian	1.936	0.151	12.821	12030	0.000	1.518	2.355	0.23
	Government - No speaker	-0.795	0.154	-5.161	12030	0.000	-1.202	-0.389	-0.09
	Government - Hanks	-0.619	0.158	-3.923	12030	0.000	-1.012	-0.225	-0.07
	Government - Kardashian	0.796	0.157	5.079	12030	0.000	0.392	1.200	0.09
	No speaker - Hanks	0.177	0.157	1.127	12030	0.260	-0.131	0.484	0.02
	No speaker - Kardashian	1.591	0.156	10.211	12030	0.000	1.154	2.029	0.19
	Hanks - Kardashian	1.415	0.159	8.880	12030	0.000	0.979	1.851	0.16
IT	Fauci - No speaker	0.256	0.120	2.138	12030	0.098	-0.031	0.543	0.04
	Fauci - Government	0.149	0.117	1.275	12030	0.405	-0.113	0.410	0.02
	Fauci - Hanks	0.886	0.121	7.340	12030	0.000	0.561	1.211	0.13
	Fauci - Kardashian	1.778	0.136	13.109	12030	0.000	1.407	2.149	0.24
	Government - No speaker	0.107	0.121	0.887	12030	0.405	-0.134	0.349	0.02

Country[1]	Comparison	MD	SE	t	df	Adj. P	LCI95	UCI95	Cohen's d
	Government - Hanks	0.737	0.122	6.050	12030	0.000	0.423	1.052	0.11
	Government - Kardashian	1.629	0.137	11.920	12030	0.000	1.250	2.008	0.22
	No speaker - Hanks	0.630	0.125	5.043	12030	0.000	0.318	0.942	0.09
	No speaker - Kardashian	1.522	0.139	10.926	12030	0.000	1.131	1.913	0.20
	Hanks - Kardashian	0.892	0.140	6.361	12030	0.000	0.522	1.261	0.12
KR	Fauci - No speaker	0.900	0.298	3.022	12030	0.023	0.074	1.726	0.06
	Fauci - Government	0.861	0.308	2.793	12030	0.037	0.031	1.691	0.05
	Fauci - Hanks	0.444	0.293	1.513	12030	0.771	-0.329	1.216	0.03
	Fauci - Kardashian	1.362	0.316	4.310	12030	0.000	0.475	2.248	0.08
	Government - No speaker	0.039	0.315	0.123	12030	0.902	-0.579	0.656	0.00
	Government - Hanks	-0.418	0.310	-1.345	12030	0.771	-1.201	0.366	-0.02
	Government - Kardashian	0.500	0.332	1.509	12030	0.771	-0.372	1.373	0.03
	No speaker - Hanks	-0.456	0.300	-1.520	12030	0.771	-1.248	0.336	-0.03
	No speaker - Kardashian	0.462	0.322	1.432	12030	0.771	-0.370	1.294	0.03
	Hanks - Kardashian	0.918	0.318	2.889	12030	0.031	0.049	1.787	0.05
US	Fauci - No speaker	0.164	0.243	0.676	12030	0.910	-0.372	0.700	0.01
	Fauci - Government	0.803	0.286	2.808	12030	0.030	0.048	1.557	0.05
	Fauci - Hanks	0.354	0.254	1.397	12030	0.515	-0.258	0.967	0.03
	Fauci - Kardashian	2.527	0.245	10.318	12030	0.000	1.848	3.206	0.19
	Government - No speaker	-0.638	0.286	-2.229	12030	0.129	-1.376	0.099	-0.04
	Government - Hanks	-0.448	0.295	-1.520	12030	0.515	-1.185	0.289	-0.03
	Government - Kardashian	1.724	0.288	5.990	12030	0.000	0.950	2.499	0.11
	No speaker - Hanks	0.190	0.254	0.747	12030	0.910	-0.380	0.760	0.01
	No speaker - Kardashian	2.363	0.245	9.639	12030	0.000	1.675	3.051	0.18
	Hanks - Kardashian	2.173	0.256	8.474	12030	0.000	1.472	2.874	0.15

[1] Country Key: BR = Brazil; CH = Switzerland; ES = Spain; IT = Italy; KR = South Korea; US = United States

Table S6. Pairwise comparisons of the spokesperson × age group interaction

Age Group	Comparison	MD	SE	t	df	Adj. P	LCI95	UCI95	Cohen's d
**Young**	Fauci - No speaker	0.499	0.121	4.113	12030	0.000	0.186	0.811	0.07
	Fauci - Government	0.435	0.127	3.437	12030	0.002	0.119	0.752	0.06
	Fauci - Hanks	0.574	0.127	4.528	12030	0.000	0.240	0.909	0.08
	Fauci - Kardashian	1.463	0.115	12.763	12030	0.000	1.145	1.781	0.23
	Government - No speaker	0.063	0.130	0.448	12030	1.000	-0.216	0.343	0.01
	Government - Hanks	0.139	0.135	1.027	12030	0.913	-0.185	0.462	0.02
	Government - Kardashian	1.028	0.124	8.316	12030	0.000	0.681	1.374	0.15
	No speaker - Hanks	0.076	0.130	0.582	12030	1.000	-0.216	0.367	0.01
	No speaker - Kardashian	0.964	0.118	8.174	12030	0.000	0.642	1.287	0.15
	Hanks - Kardashian	0.889	0.124	7.187	12030	0.000	0.556	1.221	0.13
**Mid-Age**	Fauci - No speaker	0.100	0.153	0.652	12030	0.646	-0.214	0.413	0.01
	Fauci - Government	0.582	0.158	3.680	12030	0.001	0.187	0.977	0.07
	Fauci - Hanks	0.737	0.147	5.001	12030	0.000	0.348	1.126	0.09
	Fauci - Kardashian	1.919	0.149	12.865	12030	0.000	1.511	2.327	0.23
	Government - No speaker	-0.482	0.163	-2.968	12030	0.009	-0.871	-0.093	-0.05
	Government - Hanks	0.155	0.157	0.988	12030	0.646	-0.197	0.507	0.02
	Government - Kardashian	1.337	0.159	8.401	12030	0.000	0.895	1.778	0.15
	No speaker - Hanks	0.637	0.152	4.205	12030	0.000	0.247	1.028	0.08
	No speaker - Kardashian	1.819	0.154	11.832	12030	0.000	1.388	2.251	0.22
	Hanks - Kardashian	1.182	0.148	7.977	12030	0.000	0.783	1.580	0.15
**Old**	Fauci - No speaker	0.248	0.129	1.925	12030	0.163	-0.060	0.557	0.04
	Fauci - Government	0.515	0.142	3.621	12030	0.001	0.149	0.881	0.07
	Fauci - Hanks	0.710	0.133	5.331	12030	0.000	0.359	1.062	0.10
	Fauci - Kardashian	2.168	0.151	14.341	12030	0.000	1.754	2.581	0.26
	Government - No speaker	-0.267	0.143	-1.865	12030	0.163	-0.602	0.068	-0.03
	Government - Hanks	0.195	0.147	1.324	12030	0.185	-0.094	0.484	0.02
	Government - Kardashian	1.653	0.163	10.119	12030	0.000	1.200	2.105	0.18
	No speaker - Hanks	0.462	0.134	3.436	12030	0.002	0.126	0.798	0.06
	No speaker - Kardashian	1.919	0.152	12.634	12030	0.000	1.493	2.346	0.23
	Hanks - Kardashian	1.457	0.156	9.356	12030	0.000	1.038	1.877	0.17

Table S7. Pairwise comparisons of the spokesperson × country × age group interaction

Country[1]	Age Group	Comparison	MD	SE	t	df	Adj. P	LCI95	UCI95	Cohen's d
BR	Young	Fauci - No speaker	0.442	0.121	3.480	12030	0.002	0.132	0.713	0.06
		Fauci - Government	-0.033	0.111	-0.295	12030	1.000	-0.263	0.197	-0.01
		Fauci - Hanks	0.480	0.115	4.166	12030	0.000	0.183	0.777	0.08
		Fauci - Kardashian	1.382	0.129	10.745	12030	0.000	1.025	1.739	0.20
		Government - No speaker	0.445	0.120	3.809	12030	0.001	0.157	0.754	0.07
		Government - Hanks	0.513	0.113	4.540	12030	0.000	0.215	0.811	0.08
		Government - Kardashian	1.415	0.126	11.188	12030	0.000	1.060	1.770	0.20
		No speaker - Hanks	0.058	0.123	0.469	12030	1.000	-0.219	0.334	0.01
		No speaker - Kardashian	0.960	0.136	7.075	12030	0.000	0.589	1.331	0.13
		Hanks - Kardashian	0.902	0.130	6.925	12030	0.000	0.552	1.252	0.13
	Mid-Age	Fauci - No speaker	-0.154	0.141	-1.097	12030	0.546	-0.470	0.161	-0.02
		Fauci - Government	-0.272	0.138	-1.972	12030	0.146	-0.602	0.058	-0.04
		Fauci - Hanks	0.397	0.153	2.597	12030	0.038	0.015	0.779	0.05
		Fauci - Kardashian	1.289	0.185	6.965	12030	0.000	0.783	1.795	0.13
		Government - No speaker	0.118	0.133	0.884	12030	0.546	-0.164	0.399	0.02
		Government - Hanks	0.669	0.146	4.587	12030	0.000	0.284	1.053	0.08
		Government - Kardashian	1.561	1.790	8.698	12030	0.000	1.057	2.065	0.16
		No speaker - Hanks	0.551	1.490	3.708	12030	0.001	0.168	0.934	0.07
		No speaker - Kardashian	1.443	0.182	7.934	12030	0.000	0.939	1.947	0.14
		Hanks - Kardashian	0.892	0.191	4.679	12030	0.000	0.379	1.405	0.09
	Old	Fauci - No speaker	0.076	0.183	0.418	12030	0.676	-0.282	0.435	0.01
		Fauci - Government	-0.249	0.167	-1.490	12030	0.409	-0.605	0.151	-0.03
		Fauci - Hanks	0.358	0.187	1.918	12030	0.275	-0.123	0.839	0.03
		Fauci - Kardashian	0.826	0.212	3.886	12030	0.001	0.236	1.415	0.07
		Government - No speaker	0.325	0.182	1.792	12030	0.293	-0.128	0.779	0.03
		Government - Hanks	0.607	0.186	3.272	12030	0.007	0.108	1.107	0.06

Country[1]	Age Group	Comparison	MD	SE	t	df	Adj. P	LCI95	UCI95	Cohen's d
		Government – Kardashian	1.075	0.212	5.077	12030	0.000	0.480	1.669	0.09
		No speaker - Hanks	0.282	0.200	1.410	12030	0.409	-0.186	0.749	0.03
		No speaker - Kardashian	0.749	0.224	3.341	12030	0.007	0.136	1.363	0.06
		Hanks - Kardashian	0.467	0.227	2.056	12030	0.239	-0.132	1.067	0.04
CH	Young	Fauci - No speaker	0.000	0.233	0.001	12030	1.000	-0.457	4.570	0.00
		Fauci - Government	0.082	0.216	0.381	12030	1.000	-0.435	0.599	0.01
		Fauci - Hanks	0.829	0.219	3.786	12030	0.001	0.251	1.408	0.07
		Fauci - Kardashian	1.907	0.180	10.615	12030	0.000	1.416	2.398	0.19
		Government - No speaker	-0.082	0.228	-0.360	12030	1.000	-0.560	0.396	-0.01
		Government - Hanks	0.747	0.214	3.495	12030	0.002	0.213	1.281	0.06
		Government - Kardashian	1.825	0.174	10.513	12030	0.000	1.343	2.306	0.19
		No speaker - Hanks	0.829	0.231	3.595	12030	0.002	0.235	1.423	0.07
		No speaker - Kardashian	1.907	0.194	9.812	12030	0.000	1.361	2.452	0.18
		Hanks - Kardashian	1.077	0.177	6.082	12030	0.000	0.601	1.554	0.11
	Mid-Age	Fauci - No speaker	-0.280	0.278	-1.007	12030	0.386	-0.849	0.289	-0.02
		Fauci - Government	0.347	0.266	1.302	12030	0.386	-0.250	0.944	0.02
		Fauci - Hanks	1.651	0.262	6.296	12030	0.000	0.959	2.343	0.11
		Fauci - Kardashian	2.272	0.242	9.380	12030	0.000	1.600	2.943	0.17
		Government - No speaker	-0.627	0.270	-2.319	12030	0.061	-1.274	0.020	-0.04
		Government - Hanks	1.304	0.254	5.130	12030	0.000	0.649	1.958	0.09
		Government - Kardashian	1.925	0.233	8.250	12030	0.000	1.287	2.563	0.15
		No speaker - Hanks	1.931	0.265	7.273	12030	0.000	1.216	2.645	0.13
		No speaker - Kardashian	2.552	0.246	10.387	12030	0.000	1.862	3.241	0.19
		Hanks - Kardashian	0.621	0.228	2.729	12030	0.025	0.052	1.189	0.05
	Old	Fauci - No speaker	0.018	0.327	0.056	12030	0.956	-0.623	0.659	0.00
		Fauci - Government	0.460	0.338	1.363	12030	0.519	-0.348	1.269	0.02
		Fauci - Hanks	1.795	0.337	5.334	12030	0.000	0.890	2.701	0.10

Country[1]	Age Group	Comparison	MD	SE	t	df	Adj. P	LCI95	UCI95	Cohen's d
		Fauci - Kardashian	2.812	0.313	8.993	12030	0.000	1.945	3.679	0.16
		Government - No speaker	-0.442	0.357	-1.238	12030	0.519	-1.268	0.384	-0.02
		Government - Hanks	1.335	0.366	3.647	12030	0.001	0.392	2.278	0.07
		Government - Kardashian	2.351	0.343	6.855	12030	0.000	1.413	3.289	0.12
		No speaker - Hanks	1.777	0.356	4.993	12030	0.000	8.380	2.716	0.09
		No speaker - Kardashian	2.793	0.333	8.397	12030	0.000	1.859	3.727	0.15
		Hanks - Kardashian	1.016	0.342	2.973	12030	0.012	0.162	1.870	0.05
**ES**	**Young**	Fauci - No speaker	0.114	0.220	0.516	12030	1.000	-0.363	0.590	0.01
		Fauci - Government	0.664	0.229	0.290	12030	0.022	0.061	1.268	0.01
		Fauci - Hanks	0.214	0.241	0.889	12030	1.000	-0.362	0.790	0.02
		Fauci - Kardashian	1.357	0.222	6.107	12030	0.000	0.733	1.981	0.11
		Government - No speaker	-0.551	0.228	-2.416	12030	0.079	-1.138	0.036	-0.04
		Government - Hanks	-0.450	0.248	-1.816	12030	0.278	-0.107	0.169	-0.03
		Government - Kardashian	0.693	0.230	3.012	12030	0.018	0.074	1.312	0.05
		No speaker - Hanks	0.100	0.240	0.419	12030	1.000	-0.409	0.609	0.01
		No speaker - Kardashian	1.244	0.221	5.619	12030	0.000	0.630	1.857	0.10
		Hanks - Kardashian	1.143	0.242	4.733	12030	0.000	-1.857	-0.630	0.09
	**Mid-Age**	Fauci - No speaker	0.542	0.259	2.093	12030	0.073	-0.039	1.123	0.04
		Fauci - Government	1.612	0.254	6.358	12030	0.000	0.919	2.306	0.12
		Fauci - Hanks	1.012	0.247	4.097	12030	0.000	0.360	1.664	0.07
		Fauci - Kardashian	2.326	0.237	9.828	12030	0.000	1.661	2.990	0.18
		Government - No speaker	-1.070	0.275	-3.891	12030	0.001	-1.779	-0.362	-0.07
		Government - Hanks	-0.600	0.264	-2.276	12030	0.069	-1.232	0.031	-0.04
		Government - Kardashian	0.713	0.254	2.805	12030	0.020	0.078	1.348	0.05
		No speaker - Hanks	0.470	0.269	1.747	12030	0.081	-0.057	0.997	0.03
		No speaker - Kardashian	1.783	0.259	6.873	12030	0.000	1.064	2.503	0.13
		Hanks - Kardashian	1.314	0.247	5.310	12030	0.000	0.648	1.979	0.10

Country[1]	Age Group	Comparison	MD	SE	t	df	Adj. P	LCI95	UCI95	Cohen's d
	Old	Fauci - No speaker	0.379	0.287	1.321	12030	0.559	-0.308	1.066	0.02
		Fauci - Government	1.144	0.289	3.953	12030	0.001	0.365	1.923	0.07
		Fauci - Hanks	0.339	0.298	1.136	12030	0.559	-0.340	1.018	0.02
		Fauci - Kardashian	2.127	0.316	6.722	12030	0.000	1.238	3.015	0.12
		Government - No speaker	-0.765	0.293	-2.608	12030	0.041	-1.511	-0.019	-0.05
		Government - Hanks	-0.805	0.305	-2.641	12030	0.041	-1.590	-0.020	-0.05
		Government - Kardashian	0.983	0.322	3.049	12030	0.014	0.132	1.833	0.06
		No speaker - Hanks	-0.040	0.302	-0.133	12030	0.894	-0.633	0.553	0.00
		No speaker - Kardashian	1.748	0.320	5.465	12030	0.000	0.861	2.634	0.10
		Hanks - Kardashian	1.788	0.331	5.409	12030	0.000	0.884	2.692	0.10
IT	Young	Fauci - No speaker	0.329	0.176	1.870	12030	0.123	-0.065	0.723	0.03
		Fauci - Government	-0.080	0.164	-0.489	12030	0.625	-0.402	0.241	-0.01
		Fauci - Hanks	0.765	0.173	4.428	12030	0.000	0.309	1.221	0.08
		Fauci - Kardashian	1.257	0.179	7.006	12030	0.000	0.759	1.754	0.13
		Government - No speaker	0.409	0.165	2.484	12030	0.047	0.004	0.814	0.05
		Government - Hanks	0.845	0.161	5.235	12030	0.000	0.404	1.287	0.10
		Government - Kardashian	1.337	0.168	7.942	12030	0.000	0.864	1.810	0.14
		No speaker - Hanks	0.436	0.173	2.520	12030	0.047	0.004	0.869	0.05
		No speaker - Kardashian	0.928	0.180	5.166	12030	0.000	0.445	1.412	0.09
		Hanks - Kardashian	0.492	0.177	2.780	12030	0.027	0.036	0.947	0.05
	Mid-Age	Fauci - No speaker	0.052	0.225	0.232	12030	1.000	-0.409	0.513	0.00
		Fauci - Government	-0.101	0.205	-0.492	12030	1.000	-0.542	0.340	-0.01
		Fauci - Hanks	1.144	0.224	5.098	12030	0.000	0.552	1.737	0.09
		Fauci - Kardashian	1.883	0.245	7.698	12030	0.000	1.205	2.562	0.14
		Government - No speaker	0.153	0.211	0.725	12030	1.000	-0.352	0.658	0.01
		Government - Hanks	1.245	0.210	5.922	12030	0.000	0.679	1.811	0.11
		Government - Kardashian	1.984	0.231	8.577	12030	0.000	1.334	2.633	0.16

Country[1]	Age Group	Comparison	MD	SE	t	df	Adj. P	LCI95	UCI95	Cohen's d
		No speaker - Hanks	1.092	0.231	4.736	12030	0.000	0.498	1.686	0.09
		No speaker - Kardashian	1.831	0.250	7.315	12030	0.000	1.146	2.515	0.13
		Hanks - Kardashian	0.739	0.249	2.963	12030	0.012	0.116	1.362	0.05
	**Old**	Fauci - No speaker	0.387	0.218	1.774	12030	0.304	-0.158	0.933	0.03
		Fauci - Government	0.627	0.233	2.696	12030	0.035	0.028	1.226	0.05
		Fauci - Hanks	0.749	0.226	3.314	12030	0.006	0.153	1.346	0.06
		Fauci - Kardashian	2.193	0.271	8.083	12030	0.000	1.431	2.955	0.15
		Government - No speaker	-0.240	0.245	-0.978	12030	0.656	-0.789	0.310	-0.02
		Government - Hanks	0.122	0.252	0.485	12030	0.656	-0.376	0.621	0.01
		Government - Kardashian	1.566	0.293	5.337	12030	0.000	0.764	2.369	0.10
		No speaker - Hanks	0.362	0.239	1.515	12030	0.390	-0.210	0.934	0.03
		No speaker - Kardashian	2.806	0.282	6.399	12030	0.000	1.023	2.589	0.12
		Hanks - Kardashian	1.444	0.288	5.012	12030	0.000	0.669	2.219	0.09
**KR**	**Young**	Fauci - No speaker	1.823	0.441	4.134	12030	0.000	0.585	3.061	0.08
		Fauci - Government	1.404	0.502	2.795	12030	0.047	0.011	2.796	0.05
		Fauci - Hanks	0.823	0.491	1.675	12030	0.470	-0.443	2.088	0.03
		Fauci - Kardashian	0.918	0.442	2.077	12030	0.302	-0.291	2.126	0.04
		Government - No speaker	0.419	0.529	0.793	12030	1.000	-0.797	1.635	0.01
		Government - Hanks	-0.581	0.572	-1.016	12030	1.000	-2.010	0.848	-0.02
		Government - Kardashian	-0.486	0.529	-0.918	12030	1.000	-1.739	0.767	-0.02
		No speaker - Hanks	-1.000	0.519	-1.928	12030	0.377	-2.396	0.396	-0.04
		No speaker - Kardashian	-0.905	0.472	-1.918	12030	0.377	-2.171	0.361	-0.03
		Hanks - Kardashian	0.095	0.519	0.183	12030	1.000	-0.957	1.147	0.00
	**Mid-Age**	Fauci - No speaker	0.623	0.656	0.950	12030	1.000	-0.941	2.187	0.02
		Fauci - Government	0.368	0.656	0.950	12030	1.000	-0.933	1.668	0.02
		Fauci - Hanks	-0.021	0.601	-0.034	12030	1.000	-1.206	1.165	0.00
		Fauci - Kardashian	0.921	0.613	1.502	12030	1.000	-0.743	2.586	0.03

Country[1]	Age Group	Comparison	MD	SE	t	df	Adj. P	LCI95	UCI95	Cohen's d
		Government - No speaker	0.256	0.613	0.418	12030	1.000	-1.045	1.556	0.01
		Government - Hanks	-0.388	0.553	-0.702	12030	1.000	-1.635	0.858	-0.01
		Government - Kardashian	0.554	0.566	0.977	12030	1.000	-0.806	1.913	0.02
		No speaker - Hanks	-0.644	0.626	-1.029	12030	1.000	-2.164	0.876	-0.02
		No speaker - Kardashian	0.298	0.639	0.466	12030	1.000	-1.073	1.669	0.01
		Hanks - Kardashian	0.942	0.582	1.619	12030	1.000	-0.691	2.574	0.03
	**Old**	Fauci - No speaker	0.253	0.417	0.608	12030	1.000	-0.667	1.174	0.01
		Fauci - Government	0.812	0.511	1.590	12030	0.671	-0.536	2.160	0.03
		Fauci - Hanks	0.529	0.414	1.277	12030	1.000	-0.538	1.596	0.02
		Fauci - Kardashian	2.246	0.570	3.939	12030	0.001	0.645	3.846	0.07
		Government - No speaker	-0.559	0.489	-1.144	12030	1.000	-1.778	0.660	-0.02
		Government - Hanks	-0.283	0.511	-1.590	12030	0.671	-2.160	0.536	-0.03
		Government - Kardashian	1.433	0.625	2.294	12030	0.153	-0.248	3.114	0.04
		No speaker - Hanks	0.275	0.388	0.711	12030	1.000	-0.600	1.151	0.01
		No speaker - Kardashian	1.992	0.551	3.614	12030	0.003	0.463	3.521	0.07
		Hanks - Kardashian	1.717	0.550	3.122	12030	0.014	0.213	3.221	0.06
**US**	**Young**	Fauci - No speaker	0.305	0.431	0.708	12030	1.000	-0.668	1.278	0.01
		Fauci - Government	0.576	0.432	1.334	12030	1.000	-0.563	1.715	0.02
		Fauci - Hanks	0.334	4.330	0.773	12030	1.000	-0.657	1.326	0.01
		Fauci - Kardashian	1.957	0.382	5.121	12030	0.000	0.884	3.030	0.09
		Government - No speaker	-0.271	0.426	-0.636	12030	1.000	-1.217	0.676	-0.01
		Government - Hanks	-0.241	0.427	-0.565	12030	1.000	-1.176	0.694	-0.01
		Government - Kardashian	1.381	0.376	3.669	12030	0.002	0.368	2.394	0.07
		No speaker - Hanks	0.029	0.426	0.069	12030	1.000	-0.816	0.875	0.00
		No speaker - Kardashian	1.652	0.375	4.404	12030	0.000	0.612	2.692	0.08
		Hanks - Kardashian	1.623	0.337	4.305	12030	0.000	0.592	2.653	0.08
	**Mid-Age**	Fauci - No speaker	-0.186	0.442	-0.421	12030	1.000	-1.125	0.753	-0.01

Country[1]	Age Group	Comparison	MD	SE	t	df	Adj. P	LCI95	UCI95	Cohen's d
		Fauci - Government	1.537	0.601	2.556	12030	**0.064**	-0.050	3.124	0.05
		Fauci - Hanks	0.239	0.463	0.515	12030	1.000	-0.765	1.243	0.01
		Fauci - Kardashian	2.822	0.467	6.044	12030	0.000	1.527	4.117	0.11
		Government - No speaker	-1.723	0.603	-2.859	12030	0.030	-3.345	-0.102	-0.05
		Government - Hanks	-1.298	0.618	-2.099	12030	0.179	-2.892	0.295	-0.04
		Government - Kardashian	1.285	0.622	2.065	12030	0.179	-0.300	2.869	0.04
		No speaker - Hanks	0.425	0.465	0.914	12030	1.000	-0.689	1.539	0.02
		No speaker - Kardashian	3.008	0.468	6.422	12030	0.000	1.693	4.323	0.12
		Hanks - Kardashian	2.583	0.490	5.269	12030	0.000	1.242	3.924	0.10
	**Old**	Fauci - No speaker	0.374	0.388	0.964	12030	1.000	-0.554	1.302	0.02
		Fauci - Government	0.295	0.433	0.681	12030	1.000	-0.677	1.268	0.01
		Fauci - Hanks	0.490	0.422	1.161	12030	1.000	-0.623	1.602	0.02
		Fauci - Kardashian	2.802	0.420	6.668	12030	0.000	1.622	3.983	0.12
		Government - No speaker	0.079	0.439	0.180	12030	1.000	-0.809	0.967	0.00
		Government - Hanks	0.195	0.469	0.415	12030	1.000	-0.801	1.190	0.01
		Government - Kardashian	2.507	0.467	5.365	12030	0.000	1.229	3.785	0.10
		No speaker - Hanks	0.116	4.280	0.270	12030	1.000	-0.766	0.998	0.00
		No speaker - Kardashian	2.428	0.426	5.697	12030	0.000	1.246	3.611	0.10
		Hanks - Kardashian	2.313	0.458	5.055	12030	0.000	1.082	3.544	0.09

[1] Country Key: BR = Brazil; CH = Switzerland; ES = Spain; IT = Italy; KR = South Korea; US = United States

Table S8. Model summary of the effect of sentiment towards spokesperson on message sharing

**Source**	**F**	**df1**	**df2**	**p-value[1]**	**η_p^2**	**Cohen's d**
Corrected Model	73.151	67	9,724	**0.000**	0.335	1.42
Spokesperson	46.745	3	9,724	**0.000**	0.014	0.24
Country	15.385	5	9,724	**0.000**	0.008	0.18
Attitude (towards Spokesperson)	132.648	2	9,724	**0.000**	0.027	0.33
Spokesperson × Country	2.436	15	9,724	**0.001**	0.004	0.12
Spokesperson × Attitude	2.200	6	9,724	**0.040**	0.001	0.07
Country × Attitude	4.140	10	9,724	**0.000**	0.004	0.13
Spokesperson × Attitude × Country	1.454	27	9,724	0.060	0.004	0.13

[1] p-values < 0.05 are highlighted in bold

Table S9. Pairwise comparisons of the spokesperson × likeability interaction

Attitude	Comparison	MD	SE	t	df	Adj. P	LCI95	UCI95	Cohen's d
**Like**	Fauci - Government	0.151	0.055	2.733	9724	0.013	0.027	0.274	0.055
	Fauci - Hanks	0.510	0.053	9.680	9724	0.000	0.371	0.649	0.196
	Fauci - Kardashian	0.560	0.106	5.278	9724	0.000	0.295	0.925	0.107
	Government - Hanks	0.359	0.053	6.835	9724	0.000	0.224	0.494	0.139
	Government - Kardashian	0.409	0.106	3.859	9724	0.000	0.155	0.663	0.078
	Hanks - Kardashian	0.050	0.105	0.477	9724	0.633	-0.155	0.255	0.010
**Neutral**	Fauci - Government	0.195	0.052	3.723	9724	0.000	0.078	0.313	0.076
	Fauci - Hanks	0.383	0.048	7.916	9724	0.000	0.262	0.503	0.161
	Fauci - Kardashian	0.748	0.042	17.894	9724	0.000	0.640	0.856	0.363
	Government - Hanks	0.187	0.058	3.254	9724	0.001	0.074	0.300	0.066
	Government - Kardashian	0.553	0.052	10.584	9724	0.000	0.415	0.691	0.215
	Hanks - Kardashian	0.365	0.048	7.595	9724	0.000	0.250	0.481	0.154
**Dislike**	Fauci - Government	0.235	0.130	1.804	9724	0.214	-0.077	0.547	0.037
	Fauci - Hanks	0.191	0.201	0.953	9724	0.681	-0.259	0.642	0.019
	Fauci - Kardashian	0.809	0.141	5.727	9724	0.000	0.436	1.181	0.116
	Government - Hanks	-0.043	0.179	-0.242	9724	0.809	-0.395	0.308	-0.005
	Government - Kardashian	0.574	0.109	5.291	9724	0.000	0.294	0.854	0.107
	Hanks - Kardashian	0.617	0.188	3.290	9724	0.004	0.149	1.086	0.067

Table S10. Pairwise comparisons of the country[1] × likeability interaction

Attitude	Comparison	MD	SE	t	df	Adj. P	LCI95	UCI95	Cohen's d
**Like**	BR-CH	0.397	0.088	4.492	9724	0.000	0.137	0.656	0.091
	BR-ES	0.210	0.077	2.722	9724	0.065	-0.007	0.427	0.055
	BR-IT	0.032	0.062	0.524	9724	1.000	-0.106	0.170	0.011
	BR-KR	-0.139	0.098	-1.417	9724	0.626	-0.383	0.106	-0.029
	BR-US	0.233	0.086	2.710	9724	0.000	-0.656	-0.137	0.055
	CH-ES	-0.186	0.111	-1.681	9724	0.557	-0.479	0.106	-0.034
	CH-IT	-0.364	0.100	-3.625	9724	0.004	-0.655	-0.074	-0.074
	CH-KR	-0.535	0.126	-4.249	9724	0.000	-0.902	-0.168	-0.086
	CH-US	-0.163	0.117	-1.397	9724	0.626	-0.454	0.127	-0.028
	ES-IT	-0.178	0.091	-1.958	9724	0.352	-0.423	0.067	-0.040
	ES-KR	-0.349	0.118	-2.945	9724	0.360	-0.685	-0.013	-0.060
	ES-US	0.023	0.109	0.209	9724	1.000	-0.199	0.245	0.004
	IT-KR	-0.171	0.109	-1.568	9724	0.584	-0.451	0.110	-0.032
	IT-US	0.201	0.098	2.039	9724	0.332	-0.069	0.470	0.041
	KR-US	0.372	0.124	2.988	9724	0.034	0.015	0.729	0.061
**Neutral**	BR-CH	0.486	0.034	14.274	9724	0.000	0.386	0.586	0.290
	BR-ES	0.411	0.037	11.131	9724	0.000	0.304	0.519	0.226
	BR-IT	0.169	0.032	5.345	9724	0.000	0.080	0.258	0.108
	BR-KR	0.126	0.067	1.863	9724	0.313	-0.048	0.300	0.038
	BR-US	0.413	0.069	6.030	9724	0.000	0.217	0.610	0.122
	CH-ES	-0.074	0.043	-1.735	9724	0.331	-0.181	0.033	-0.035
	CH-IT	-0.317	0.038	-8.273	9724	0.000	-0.427	-0.206	-0.168
	CH-KR	-0.360	0.071	-5.081	9724	0.000	-0.557	-0.163	-0.103
	CH-US	-0.072	0.072	-1.006	9724	0.944	-0.244	0.100	-0.020
	ES-IT	-0.242	0.041	-5.926	9724	0.000	-0.359	-0.126	-0.120
	ES-KR	-0.286	0.072	-3.952	9724	0.001	-0.484	-0.088	-0.080
	ES-US	0.002	0.073	0.027	9724	1.000	-0.142	0.146	0.001
	IT-KR	-0.043	0.070	-0.622	9724	1.000	-0.200	0.113	-0.013
	IT-US	0.244	0.071	3.453	9724	0.004	0.054	0.435	0.070
	KR-US	0.288	0.093	3.109	9724	0.011	0.044	0.532	0.063
**Dislike**	BR-CH	0.560	0.170	3.297	9724	0.012	0.073	1.047	0.067
	BR-ES	0.613	0.147	4.171	9724	0.000	0.185	1.042	0.085
	BR-IT	0.653	0.172	3.789	9724	0.002	0.155	1.152	0.077
	BR-KR	1.037	0.198	5.227	9724	0.000	0.454	1.619	0.106
	BR-US	0.596	0.281	2.123	9724	0.304	-0.183	1.374	0.043
	CH-ES	0.053	0.149	0.357	9724	1.000	-0.260	0.367	0.007
	CH-IT	0.093	0.174	0.535	9724	1.000	-0.367	0.554	0.011
	CH-KR	0.477	0.200	2.381	9724	0.190	-0.091	1.045	0.048
	CH-US	0.036	0.282	0.126	9724	1.000	-0.530	0.601	0.003

Attitude	Comparison	MD	SE	t	df	Adj. P	LCI95	UCI95	Cohen's d
	ES-IT	0.040	0.152	0.262	9724	1.000	-0.274	0.354	0.005
	ES-KR	0.423	0.181	2.336	9724	0.195	-0.085	0.932	0.047
	ES-US	-0.018	0.268	-0.066	9724	1.000	-0.551	0.515	-0.001
	IT-KR	0.383	0.202	1.894	9724	0.466	-0.170	0.937	0.038
	IT-US	-0.058	0.283	-0.204	9724	1.000	-0.634	0.519	-0.004
	KR-US	-0.441	0.300	-1.471	9724	0.989	-1.248	0.366	-0.030

[1] Country Key: BR = Brazil; CH = Switzerland; ES = Spain; IT = Italy; KR = South Korea; US = United States

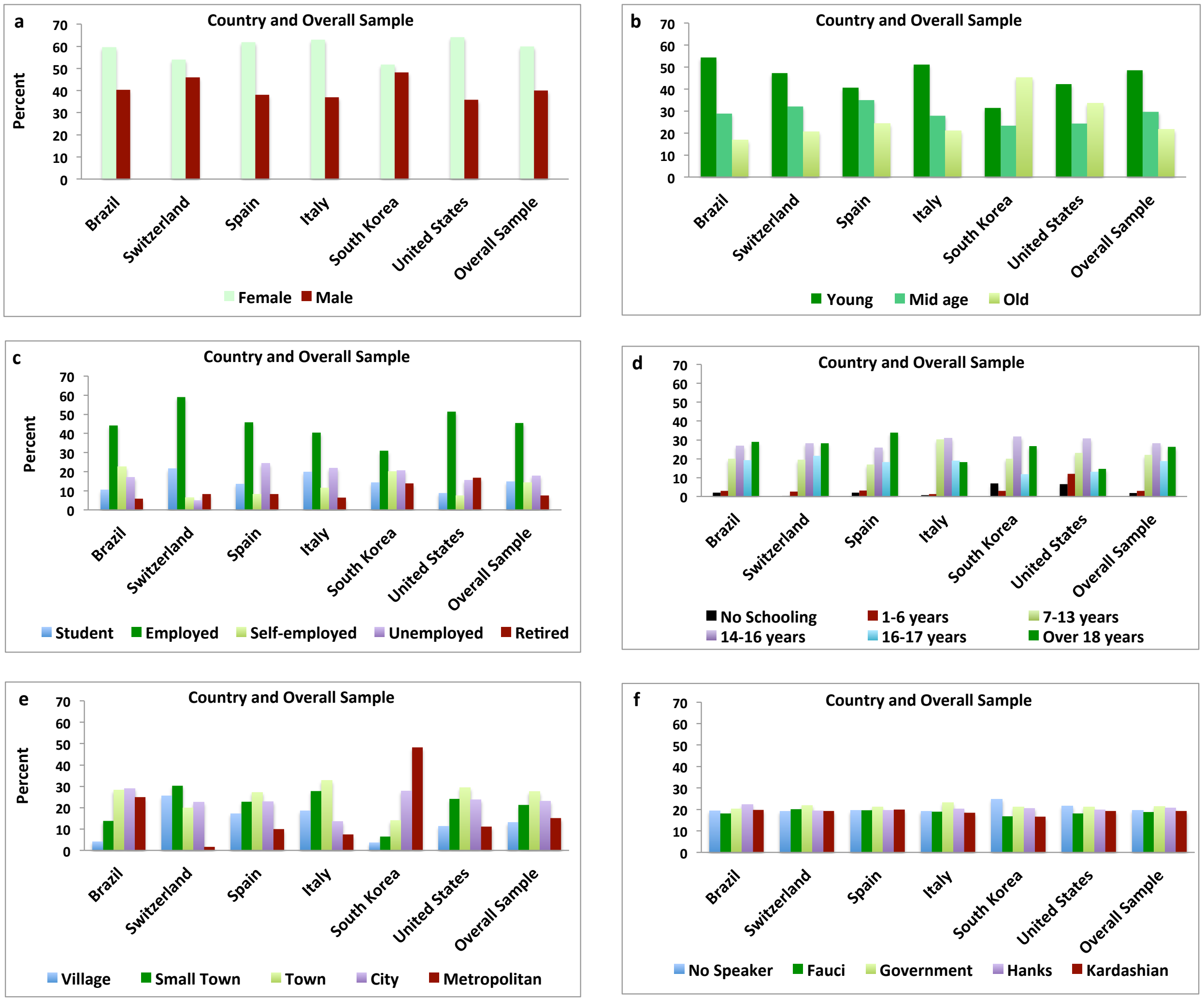

Fig. S1. Sample characteristics by country and overall sample in terms of (**a**) gender, (**b**) age group, (**c**) employment, (**d**) education, (**e**) settlement size, and (**f**) spokesperson distribution.

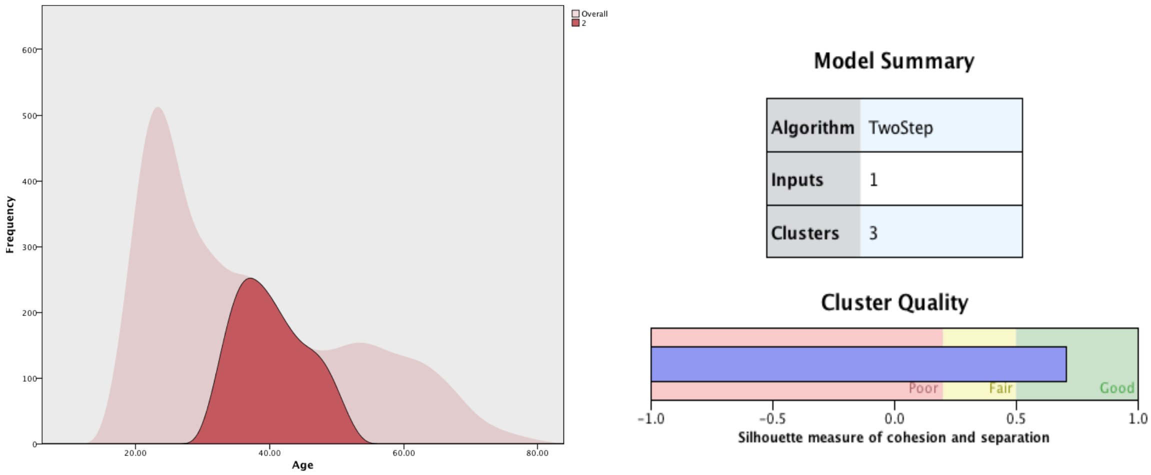

Fig. S2. Two-step cluster analysis of age. Left panel displays the density plot of age, highlighting in burgundy, the mid-age group. Right panel displays the silhouette measure of cluster quality.

Endorsement of social and physical distancing measures

We surveyed nine measures: (i) Self-isolation / staying at home, (ii) Quarantine zones that no-one can enter or leave, (iii) Closing international borders, (iv) Not meeting friends and family, (v) Keeping physical distance (2m / 6ft) at all times, (vi) Closing schools, universities, daycares and non-essential businesses, (vii) Shutting down flights and public transportation, (viii) Canceling events and public gatherings, and (ix) Not going to your place of work. Response reliability to the nine measures was good (Cronbach's α = 0.81).

At the spokesperson level (Fig. S1), respondents indicated the highest overall endorsement of physical and social distancing measures under the Fauci condition (Mean = 74.5%, 95% CI = 73.9% — 75.2%), and the lowest under the No speaker condition (Mean = 71.6%, 95% CI = 70.9% — 72.2%). At the country level, respondents from South Korea indicated the lowest overall endorsement (Mean = 53.6%, 95% CI = 48.8% — 58.4%), and respondents from Spain indicated the highest overall endorsement (Mean = 83.9%, 95% CI = 80% — 87.4%). With respect to individual measures, quarantine zones, transportation, not going to the workplace, not meeting friends and family, and closing borders received lower levels of endorsement (range 55% [quarantine zones] – 70% [closing borders]). On the other hand, self isolation, physical distance, closing businesses and canceling events received higher levels of endorsement (range 80% [physical distancing] – 89% [canceling events]).

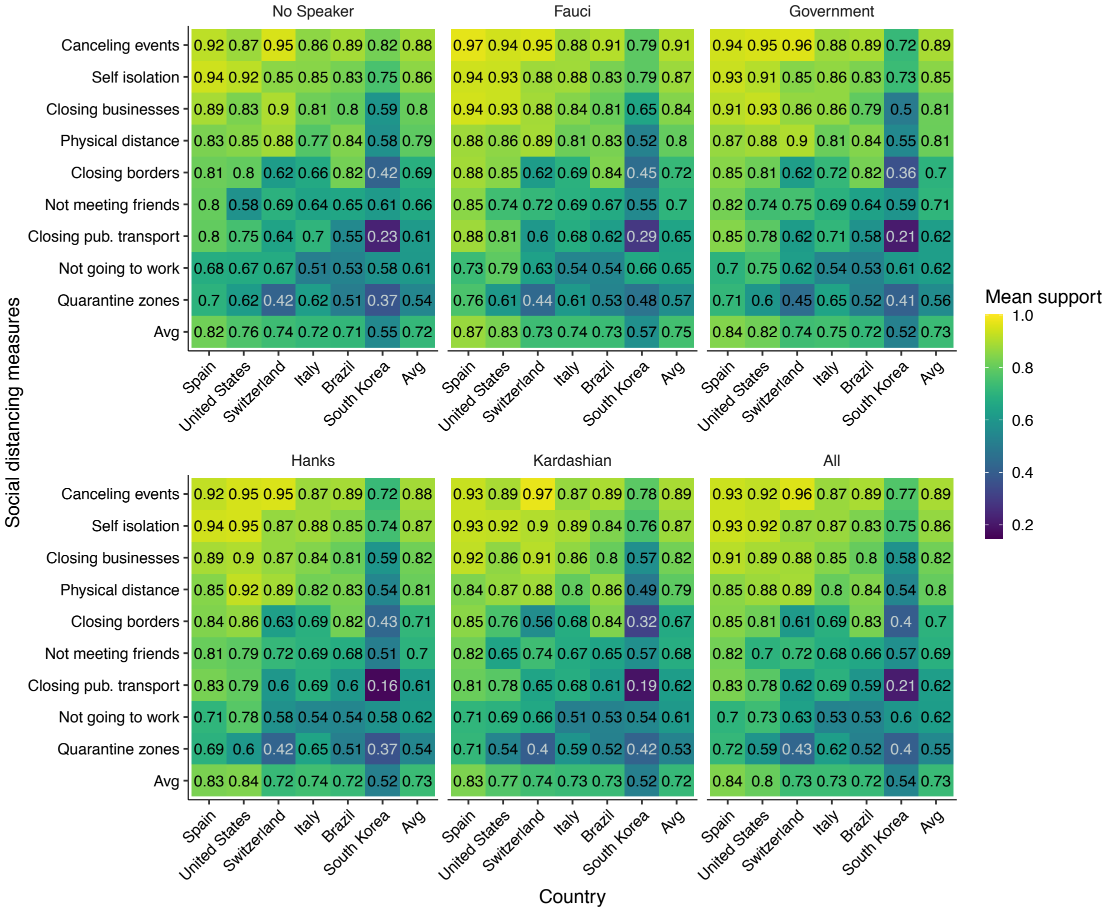

Fig. S3. Endorsement of individual distancing measures. Heatmap of mean endorsement of nine social/physical distancing measures by spokesperson and country, and for the overall sample.

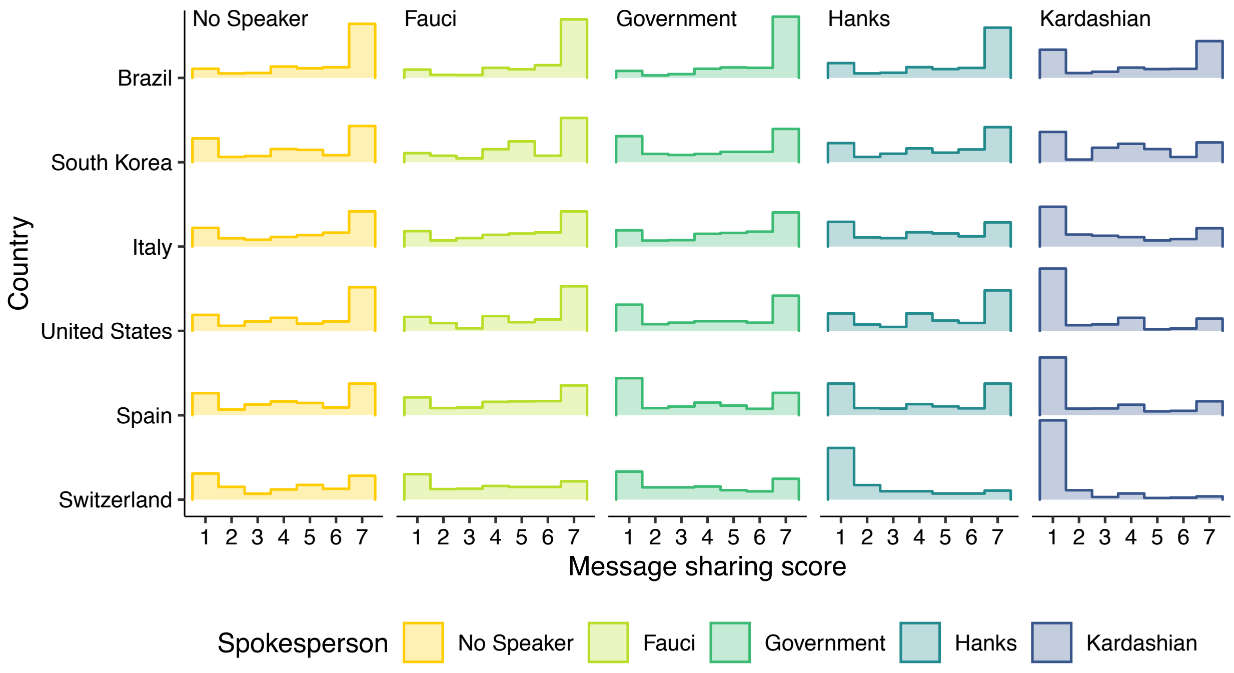

Fig. S4. Frequency of message sharing by country and spokesperson as a ridge frequency plot

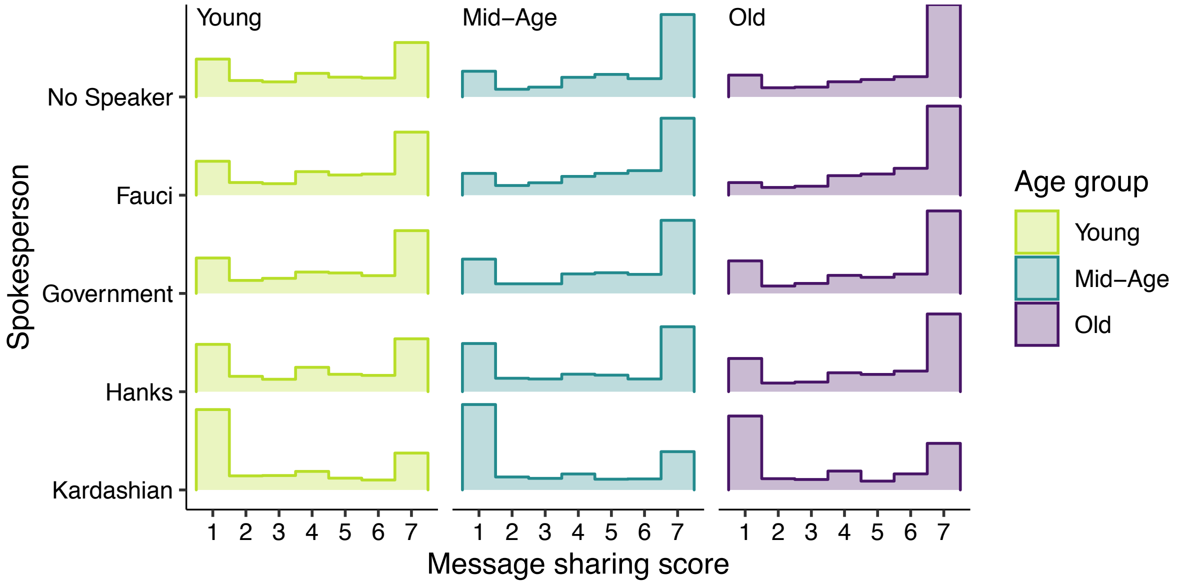

Fig. S5. Frequency of message sharing by spokesperson and age group as a ridge frequency plot

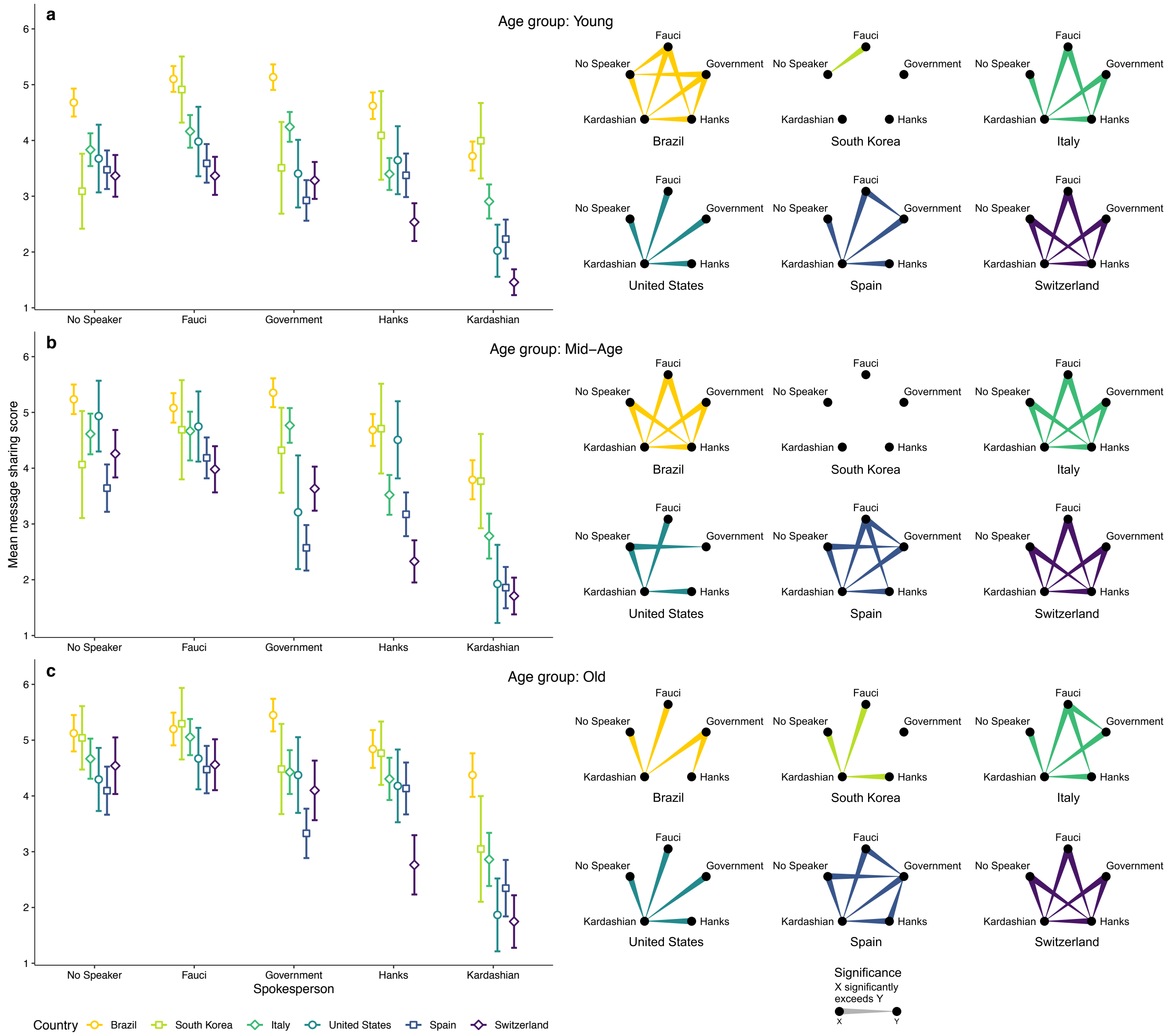

Fig. S6. Three-way interaction of spokesperson × country × age group. Panels **a-c** visualize the interaction of spokesperson x country among the young, mid-age and older respondents, with corresponding spokesperson pairwise comparisons within each country, corrected for multiple comparisons. Only significant comparisons surviving correction are shown. Error bars represent 95% CIs.

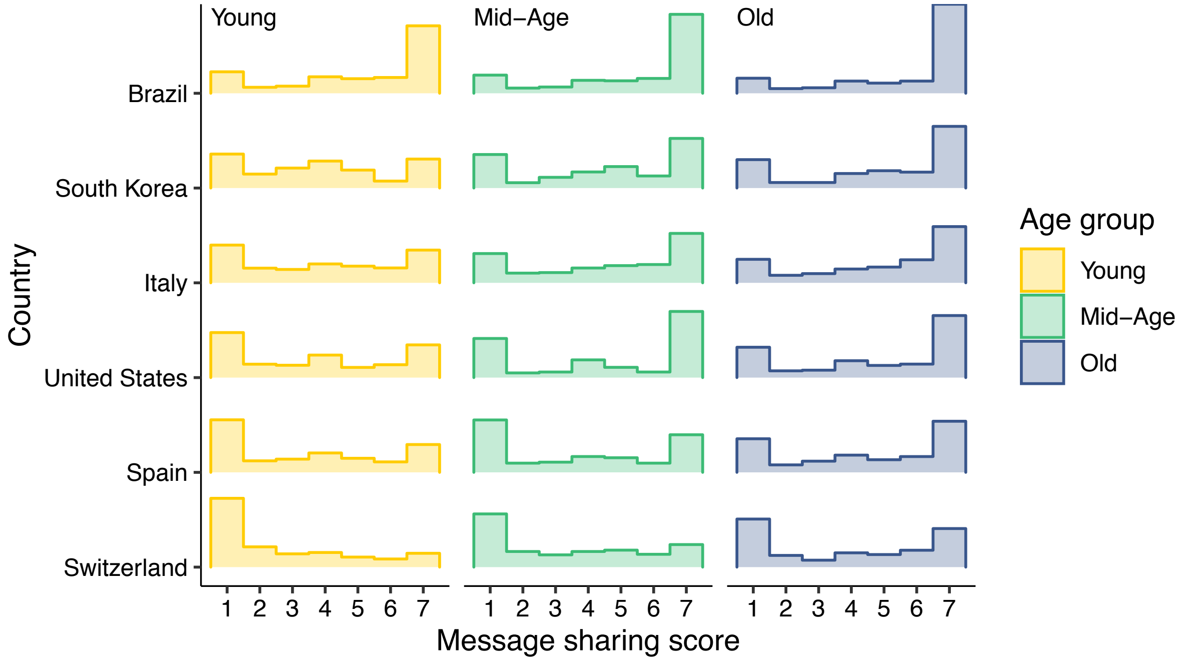

Fig. S7. Frequency of message sharing by country and age group as a ridge frequency plot

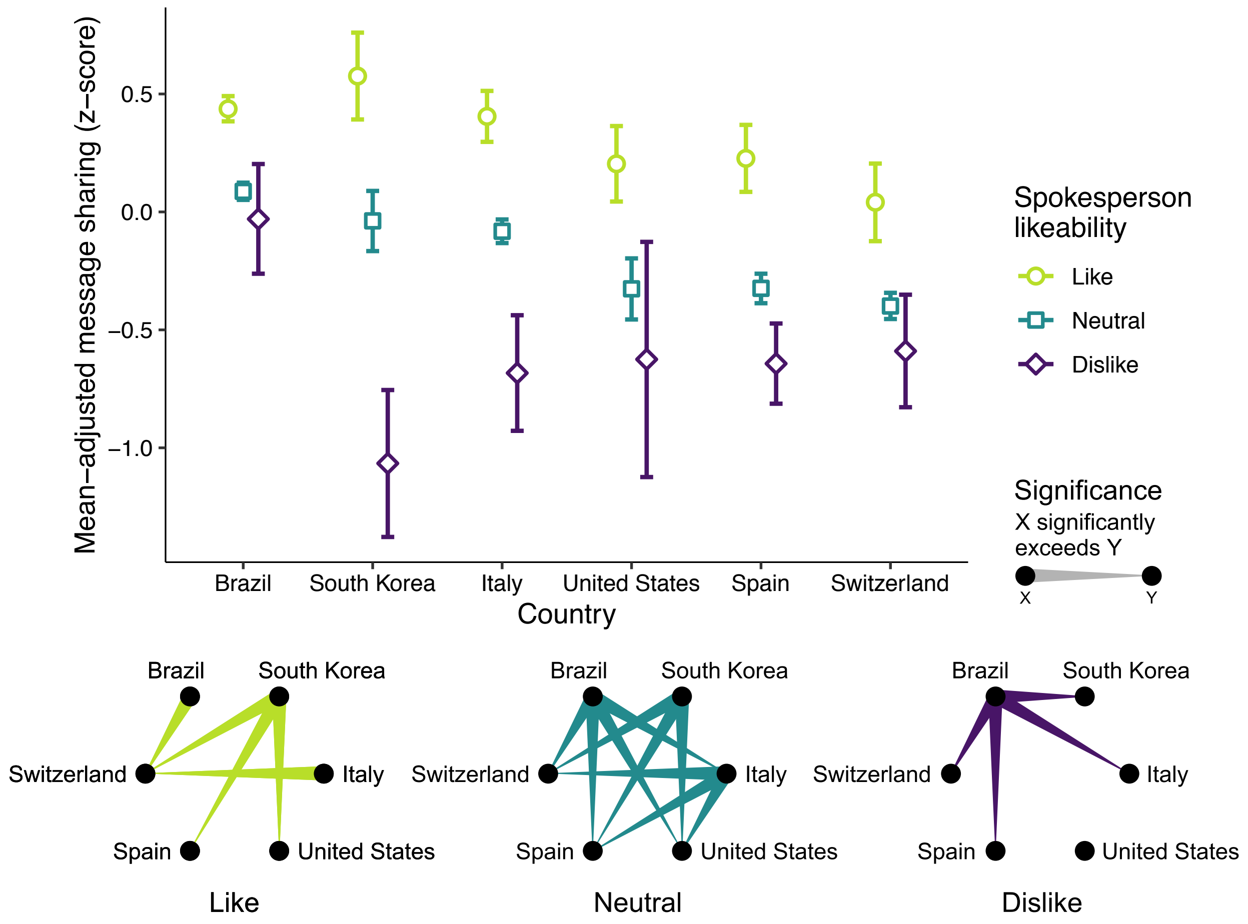

Fig. S8. Interaction of country × likeability. Pairwise comparisons of country for each sentiment category (Like, Neutral, Dislike) show only significant comparisons that survived correction for multiple testing. Error bars represent 95% CIs.

Appendix A: Study survey*

If you have 2-3 minutes, we would greatly appreciate it if you could take this short survey to tell us how the spread of coronavirus disease (COVID-19) is affecting your life. We are a university research lab trying to better understand how people are dealing with the crisis. Your input matters!

This survey is anonymous.

**Q1**	How worried are you about the COVID-19 situation in <country> right now?	(1) I am not worried<br>(7) I am extremely worried
**Q2**	Please share your opinion about the response of the <country adj.> government and of the <country adj.> population to COVID-19.	Text input

In an effort to avoid spreading COVID-19, a commonly given instruction is to practice SOCIAL DISTANCING, that is, to deliberately stay away from other people by at least 2 meters (6 feet).

Examples of social distancing are
- canceling sports events, cruises, festivals and other gatherings,
- working from home instead of at the office,
- closing schools and universities or switching to online classes,
- visiting loved ones by electronic devices instead of in person,
- canceling or postponing conferences and large meetings.

<image of speaker / no image>

Social distancing has been publicly supported, among others, by <speaker>.

**Q3**	How likely is it that you would share this message by <speaker> on your own social media?	(1) Very unlikely<br>(7) Very likely
**Q4**	Were you aware of the instruction to practice social distancing?	Yes / No
**Q5**	To what degree do you support social distancing as a valid measure in the current situation?	(1) I don't support it<br>(7) I fully support it
**Q6**	Given the current situation, which measures of social distancing do you find appropriate? (Please select all that apply)	* Self-isolation / staying at home<br>* Quarantine zones that no-one can enter or leave<br>* Closing international borders<br>* Not meeting friends and family<br>* Keeping physical distance (2m / 6ft) at all times<br>* Closing schools, universities, daycares and non-essential businesses<br>* Shutting down flights and public transportation<br>* Canceling events and

		public gatherings<br>* Not going to your place of work
**Q7**	To what degree are you currently practicing social distancing?	(1) Not at all<br>(7) All the time
**Q8**	To what degree do you think others are currently practicing social distancing?	(1) Not at all<br>(7) All the time
**Q9**	To what degree do you see yourself practicing social distancing in the weeks to come?	(1) Not at all<br>(7) All the time
**Q10**	How do you feel about <speaker>?	* I like <speaker><br>* I neither like nor dislike <speaker><br>* I dislike <speaker><br>* (I don't know <speaker>)
**Q11**	What is your personal estimate of the percentage of people in your place of residence (city/town/village) who are actually already infected by coronavirus? (Give your best personal guess of the percentage of *actually* infected people (tested + untested), not the official statistics of people who tested positive.)	0-9%<br>10-19%<br>...<br>90-100%
**Q12**	How concerned are you for the well-being of your fellow citizens at the current time?	(1) Not at all<br>(7) Very concerned
**Q13**	How would you rate your overall health in the last 30 days?	Very good<br>Good<br>Average<br>Bad<br>Very bad

Considering the current situation in <country>, please state the level to which you agree with the following statements.

**Q14**	"I feel free to move around and travel wherever I need to in order to go about my daily life, to attend appointments or to visit family or friends."	(1) Disagree strongly<br>(7) Agree strongly
**Q15**	"I am satisfied with the <country adj.> government's effort and preparedness to fight COVID-19."	(1) Disagree strongly<br>(7) Agree strongly
**Q16**	"I think the <country adj.> government cares more about public health than about the economy."	(1) Disagree strongly<br>(7) Agree strongly

Please share some details about yourself.

**Q17**	Gender	Female / Male / Other
**Q18**	Age	Numerical input

**Q19**	How many years (full-time equivalent) have you been in formal education? Include all primary and secondary schooling, university and other post-secondary education, and full-time vocational training, but do not include repeated years. If you are currently in education, count the number of years you have completed so far.	I have no formal schooling<br>1-6 years<br>7-13 years<br>14-16 years<br>17-18 years<br>More than 18 years
**Q20**	What is your current employment status?	Student<br>Employed<br>Self-employed<br>Unemployed<br>Retired
**Q21**	What is your current country of residence?	Text input
**Q22**	Which of the following best describes the area in which you live?	* Village / rural area (fewer than 3,000 people)<br>* Small town (3,000 to 15,000 people)<br>* Town (15,000 to 100,000 people)<br>* City (100,000 to 1,000,000 people)<br>* Metropolitan area (over 1,000,000 people)
**Q23**	How many people live in your household or shared apartment (including you)?	Numerical input
**Q24**	How important is religion in your daily life?	(1) Not important at all<br>(7) Very important

* The survey was administered in the following countries and languages: Brazil (Portuguese), Italy (Italian), Spain (Spanish), South Korea (Korean), Switzerland (French, German, Italian), and United States (English). All localized versions were translated from this English original by native speakers.

Appendix B: Web Sources for Spokesperson statements of support

For all spokespersons, we ensured their public and outspoken support of social distancing measures prior to their inclusion in the survey. The following is a list of Web sources of these statements.

Country	Spokesperson	Source
US	Donald Trump, President	https://twitter.com/realDonaldTrump/status/1238824050924883968
CH	Simonetta Sommaruga, President of the Swiss Confederation	https://twitter.com/s_sommaruga/status/1241265194392793088
IT	Giuseppe Conte, Prime Minister	https://twitter.com/GiuseppeConteIT/status/1237863027254243333
ES	Pedro Sánchez, Prime Minister	https://www.nytimes.com/2020/03/15/world/europe/spain-coronavirus.html
BR	Luiz Henrique Mandetta, Minister of Health*	https://twitter.com/minsaude/status/1240753287491727361
KR	Moon Jae-in, President	https://twitter.com/TheBlueHouseENG/status/1239742815141105665
Celebrity	Tom Hanks	https://twitter.com/tomhanks/status/1241919151829954566
Celebrity	Kim Kardashian	https://twitter.com/KimKardashian/status/1242202609420730374
Expert	Anthony Fauci	https://www.today.com/video/dr-anthony-fauci-social-distancing-will-likely-continue-for-at-least-several-weeks-81011269698

*The President of Brazil, Jair Bolsonaro, did not issue public support of social distancing. In his place, we included Minister Luiz Henrique Mandetta as the Brazilian government official, who was the Minister of Health at the time of the survey.

Appendix C: Timeline of government issuance of key social distancing measures

To establish timelines of implemented measures for all countries in which we distributed the survey, we collected official government announcements for nation-wide action, or news articles reporting on these announcements in cases where we could not locate the formal announcements.

Country	Measure	Description	Date Announced	Source
Brazil	Advice to self-isolate when sick	Patients with respiratory symptoms and their family members are required to self-quarantine for 14 days	2020-03-20	https://www.saude.gov.br/noticias/agencia-saude/46568-ministerio-da-saude-declara-transmissao-comunitaria-nacional
	Physical distancing encouraged	The population is advised to avoid crowding	2020-03-13	https://www.saude.gov.br/noticias/agencia-saude/46540-saude-anuncia-orientacoes-para-evitar-a-disseminacao-do-coronavirus
	Public events banned	Not implemented*		
	Public gatherings banned	Not implemented*		
	School closure ordered	Not implemented*		
	Non-essential business closure	Not implemented*		
	International border closing	The border to Venezuela is closed except for cargo	2020-03-17	https://www.reuters.com/article/us-health-coronavirus-brazil-venezuela-idUSKBN2143ZS
	Quarantine zones established	Not implemented		
Italy	Advice to self-isolate when sick	Advice to self-isolate with fever and respiratory symptoms	2020-03-08	http://www.salute.gov.it/portale/nuovocoronavirus/dettaglioNotizieNuovoCoronavirus.jsp?id=4175
	Physical distancing encouraged	Citizens are advised to keep at least 1m distance at all times	2020-03-04	http://www.salute.gov.it/portale/nuovocoronavirus/dettaglio

				NotizieNuovoCoronavirus.jsp?id=4156
	Public events banned	Public events are canceled country-wide	2020-03-08	http://www.salute.gov.it/portale/nuovocoronavirus/dettaglioNotizieNuovoCoronavirus.jsp?id=4175
	Public gatherings banned	Citizens are advised to stay at home unless for work- or health-related reasons. Gatherings are banned both outdoors and indoors	2020-03-09	http://www.salute.gov.it/portale/nuovocoronavirus/dettaglioNotizieNuovoCoronavirus.jsp?id=4184
	School closure ordered	Schools and universities are closed	2020-03-04	http://www.salute.gov.it/portale/nuovocoronavirus/dettaglioNotizieNuovoCoronavirus.jsp?id=4154
	Non-essential business closure	Commercial activity with the exception of basic necessities and pharmacies is ceased	2020-03-11	http://www.salute.gov.it/portale/nuovocoronavirus/dettaglioNotizieNuovoCoronavirus.jsp?id=4212
	International border closing	All travel within the country and entering the country is restricted	2020-03-09	http://www.salute.gov.it/portale/nuovocoronavirus/dettaglioNotizieNuovoCoronavirus.jsp?id=4184
	Quarantine zones established	Local quarantine zones around 12 towns in Lombardy and Veneto are established	2020-02-22	https://www.lastampa.it/milano/2020/02/23/news/coronavirus-nessun-blindato-nella-zona-rossa-del-lodigiano-ma-vigilanza-diffusa-dei-carabinieri-e-unita-mobile-coi-medici-dell-arma-1.38506501
Korea	Advice to self-isolate when sick	Citizens are advised to stay at home if they have respiratory symptoms	2020-02-23	https://www.mohw.go.kr/eng/nw/nw0101vw.jsp?PAR_MENU_ID=1007&MENU_ID=100701&page=3&CONT_SEQ=353124

	Physical distancing encouraged	Population is advised to practice social distancing	2020-02-29	https://www.cdc.go.kr/board/board.es?mid=a30402000000&bid=0030&act=view&list_no=366406
	Public events banned	Not implemented*		
	Public gatherings banned	Citizens are advised to stay at home as much as possible and minimize all interpersonal contact	2020-03-22	https://www.cdc.go.kr/board/board.es?mid=a30402000000&bid=0030&act=view&list_no=366627&tag=&nPage=1
	School closure ordered	Reopening of schools and kindergartens for the new semester is postponed	2020-03-10	https://www.mohw.go.kr/eng/nw/nw0101vw.jsp?PAR_MENU_ID=1007&MENU_ID=100701&page=1&CONT_SEQ=353522
	Non-essential business closure	Not implemented*		
	International border closing	Not implemented		
	Quarantine zones established	Not implemented		
Spain	Advice to self-isolate when sick	Citizens with respiratory symptoms and fever are advised to stay at home	2020-03-09	https://www.mscbs.gob.es/gabinete/notasPrensa.do?id=4806
	Physical distancing encouraged	Working remotely and keeping distance is encouraged	2020-03-09	https://www.mscbs.gob.es/gabinete/notasPrensa.do?id=4806
	Public events banned	Public events with over 1000 participants are canceled in the most severely affected areas	2020-03-10	https://www.mscbs.gob.es/gabinete/notasPrensa.do?id=4807
	Public gatherings banned	Nationwide lockdown is put into effect	2020-03-14	https://www.lamoncloa.gob.es/lang/en/presidente/news/Paginas/2020/20200313

				_emergency.aspx
	School closure ordered	Nationwide closure of schools	2020-03-12	https://elpais.com/sociedad/2020-03-12/suspendidas-las-clases-en-todos-los-centros-educativos-de-euskadi.html
	Non-essential business closure	Workers in non-essential sectors are ordered to stay at home	2020-03-28	https://www.lamoncloa.gob.es/lang/en/presidente/news/Paginas/2020/20200328non-essential-act.aspx
	International border closing	Borders are closed, except for residents and cargo	2020-03-16	http://www.interior.gob.es/es/web/interior/noticias/detalle/-/journal_content/56_INSTANCE_1YSSI3xiWuPH/10180/11634808/
	Quarantine zones established	Not implemented		
Switzerland	Advice to self-isolate when sick	Advice to self-isolate at home with fever or cough.	2020-02-27	https://www.bag.admin.ch/bag/de/home/das-bag/aktuell/medienmitteilungen.msg-id-78273.html
	Physical distancing encouraged	The population is advised to avoid all unnecessary contact	2020-03-16	https://www.bag.admin.ch/bag/de/home/das-bag/aktuell/medienmitteilungen.msg-id-78454.html
	Public events banned	Public events over 1000 participants are banned. Smaller events require municipal approval. This limit was reduced to 100 participants on 2020-03-13	2020-02-28	https://www.bag.admin.ch/bag/de/home/das-bag/aktuell/medienmitteilungen.msg-id-78289.html
	Public gatherings banned	Gatherings of more than 5 persons are banned	2020-03-20	https://www.bag.admin.ch/bag/de/home/das-

				bag/aktuell/medienmitteilungen.msg-id-78513.html
	School closure ordered	In-person teaching at schools and universities is suspended	2020-03-13	https://www.bag.admin.ch/bag/de/home/das-bag/aktuell/medienmitteilungen.msg-id-78437.html
	Non-essential business closure	Closure of all non-essential businesses	2020-03-16	https://www.bag.admin.ch/bag/de/home/das-bag/aktuell/medienmitteilungen.msg-id-78454.html
	International border closing	Closure of borders for everyone except residents, cargo, and transitory travel	2020-03-16	https://www.bag.admin.ch/bag/de/home/das-bag/aktuell/medienmitteilungen.msg-id-78454.html
	Quarantine zones established	Not implemented		
US	Advice to self-isolate when sick	Citizens are advised to stay at home if they are sick or not feeling well	2020-03-11	https://www.whitehouse.gov/briefings-statements/remarks-president-trump-address-nation/
	Physical distancing encouraged	Citizens are advised to keep physical distance	2020-03-18	https://twitter.com/CDCgov/status/1240057093526339593
	Public events banned	Not implemented*		
	Public gatherings banned	Citizens are advised to avoid groups of more than 10 persons	2020-03-16	https://www.whitehouse.gov/briefings-statements/remarks-president-trump-vice-president-pence-members-coronavirus-task-force-press-briefing-3/
	School closure ordered	Not implemented*		

	Non-essential business closure	Not implemented*		
	International border closing	Travel to and from Europe is suspended.	2020-03-11	https://www.whitehouse.gov/briefings-statements/remarks-president-trump-address-nation/
	Quarantine zones established	Not implemented		

* No action was announced or taken by the national (federal) government, but there were responses to this effect on a local, city, or state level

Appendix D: Facebook Advertisement

We include one possible example layout for the Facebook advertising campaign that we used to publicize the survey. Advertisements in all languages used the same image and translations of the English texts in the screenshot below. LEARN MORE redirected respondents to a survey form for their language (with a randomized spokesperson). Note that the actual layout that a respondent would have seen is one of dozens of possible options that depend on the used Facebook platform, browser, system, and window size.

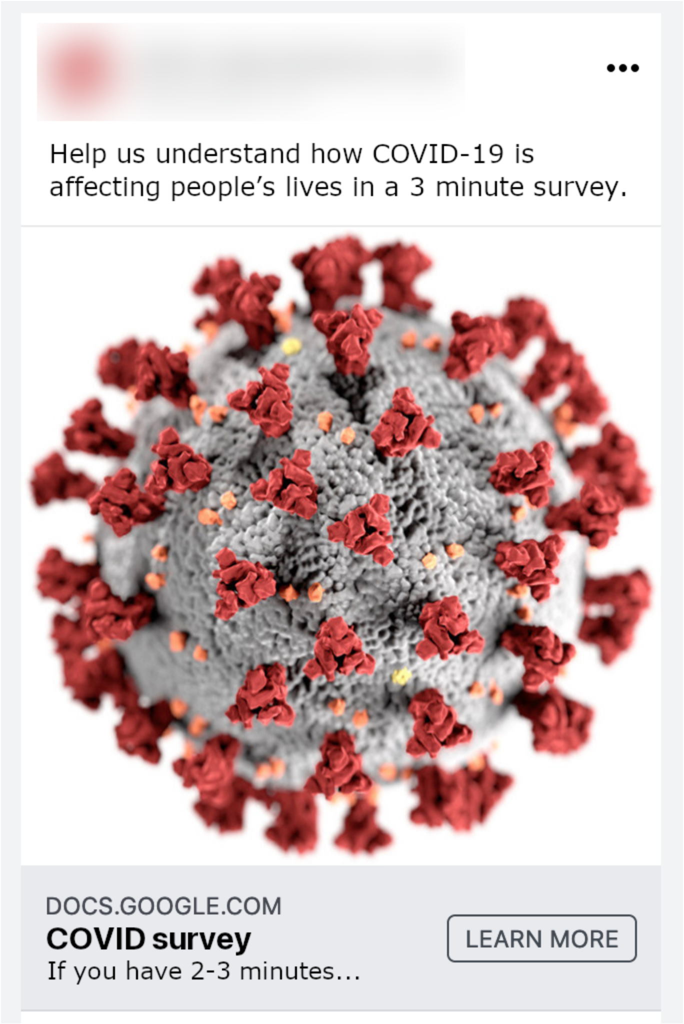

Supplement: S1 File — (PDF) [file pone.0245100.s001.pdf]
